# Single-cell RNA sequence analysis reveals USP32 as a therapeutic target to mitigate PD-L1-driven colorectal tumorigenesis *in vitro* and *in vivo*

**DOI:** 10.7150/thno.117900

**Published:** 2026-01-01

**Authors:** Girish Birappa, Haribalan Perumalsamy, Seok-Ho Hong, D. A. Ayush Gowda, Arun Pandian Chandrasekaran, Janardhan Keshav Karapurkar, Sripriya Rajkumar, Sri Renukadevi Balusamy, Aparna Jayachandran, Kwang-Hyun Baek, Junwon Lee, Viswanathaiah Matam, Woo Jin Kim, Kye-Seong Kim, Suresh Ramakrishna, Bharathi Suresh

**Affiliations:** 1 Graduate School of Biomedical Science and Engineering, Hanyang University, Seoul, 04763, South Korea.; 2 Center for Creative Convergence Education, Hanyang University, Seoul 04763, Republic of Korea.; 3 Research Institute for Convergence of Basic Science, Hanyang University, Seoul 04763, Republic of Korea.; 4 Department of Internal Medicine, College of Medicine, Kangwon National University, Chuncheon, Republic of Korea.; 5 Department of Food Science and Biotechnology, Sejong University, Gwangjin-gu, Seoul 05006, Republic of Korea.; 6 Fiona Elsey Cancer Research Institute, VIC, Australia.; 7 Federation University, VIC, Australia.; 8 Department of Biomedical Science, CHA University, Gyeonggi-Do 13488, Republic of Korea.; 9 Department of Ophthalmology, Institute of Vision Research, Gangnam Severance Hospital, Yonsei University College of Medicine, Seoul, South Korea; 10 Department of Biotechnology, Alliance School of Applied Engineering, Alliance University, Bengaluru 562106, India.; 11 Department of Internal Medicine, Kangwon National University Hospital, Chuncheon, Republic of Korea.; 12 College of Medicine, Hanyang University, Seoul, 04763, South Korea.

**Keywords:** cancer progression, deubiquitinase, polyubiquitination, prognostic marker, protein abundance, protein degradation, protein turnover, transcriptomic analysis

## Abstract

**Background:** The expression levels of the programmed death-ligand 1 (PD-L1) protein serves as a prognostic indicator for patients with colorectal cancer (CRC). Advancement of CRC is facilitated by deubiquitinating enzymes (DUBs), which regulate oncoprotein levels via the ubiquitin-proteasomal pathway. The post-translational regulatory mechanisms governing PD-L1 protein abundance on CRC, in relation to different tumor grades and their clinical relevance, remains unknown.

**Methods:** We analyzed single-cell RNA sequencing (scRNA-seq) data to identify DUB genes associated with PD-L1 expression in CRC. We used a loss-of-function-based CRISPR/Cas9 library to identify putative DUB genes that regulate the PD-L1 protein level. Immunoprecipitation was used to confirm the interaction between the USP32 and PD-L1 along with its ubiquitination status. A series of *in vitro* and *in vivo* carcinogenesis-related experiments were conducted to determine the clinical relevance between USP32 and PD-L1 expression in CRC progression.

**Results:** In this study, we analyzed scRNA-seq data from extensive cohorts of human and mice at the single-cell level to identify DUB genes associated with PD-L1 expression in CRC. Our analysis identified multiple putative DUBs, including USP32 and USP12, as prognostic markers associated with PD-L1 expression, which was found to be elevated in T cells, macrophages, and classical monocytes cell types in patients with CRC. A secondary screening using CRISPR/Cas9-mediated loss-of-function analysis for DUBs found that USP32 modulates PD-L1 protein levels in CRC. Furthermore, we demonstrated that USP32 interacts with, stabilizes, and extends the half-life of PD-L1 by preventing its K-48-linked polyubiquitination as an underlying mechanism that contributes for tumorigenesis.

**Conclusion:** A combination of scRNA-seq analysis and wet-lab experimental validation confirmed that USP32 mediates PD-L1 protein stabilization in colon cancer, identifying it as a potential therapeutic target for CRC. CRISPR/Cas9-mediated targeted knockout of the *USP32* gene reduced PD-L1 protein levels and significantly mitigated colorectal cell proliferation and tumorigenesis, both *in vitro* and *in vivo*, in a xenograft mouse model, underscoring a novel and alternative approach to the treatment of CRC.

## Introduction

Colorectal cancer (CRC) is one of the most prevalent cancers leading to death globally and is becoming more common in younger populations 1. Although chemotherapy is the principal treatment modality for most CRC patients, it comes with significant side effects, and despite its initial efficacy, chemo-resistance remains the primary obstacle in the treatment of CRC 2. CRC tumors can escape from immune surveillance, which significantly diminishes the effectiveness of chemotherapy. In CRC, the tumor microenvironment (TME) consists of neoplastic cells, stromal cells, the extracellular matrix, and diverse cell types, including fibroblasts, immune cells, and endothelial cells, all of which play critical roles in tumor development, invasion, and metastasis 3. Recently, immunotherapy has demonstrated substantial efficacy and become an alternative treatment for CRC. Immune-checkpoint inhibitors, such as programmed cell-death 1 (PD-1) and programmed death-ligand 1 (PD-L1) inhibitors, have emerged as a breakthrough approach to treatment demonstrating significant inhibitory efficacy across various cancers, including CRC 4.

Immune cells such as T-cells and macrophages express PD-1, which is a T-cell co-suppressor receptor. Its ligand is PD-L1, a transmembrane glycoprotein also known as cluster differentiation 274 (CD274), that is expressed primarily on dendritic cells and various types of tumor cells. PD-L1 inhibits activated T-cells by interacting with PD-1 to protect the host from autoimmune diseases 5. However, as cancer spreads, PD-L1 expression increases on tumor surfaces, making it easier to identify and engage with T lymphocytes and PD-1, thereby obstructing immune surveillance 6-8. Multiple studies have demonstrated that elevated PD-L1 expression in CRC correlates with a reduced survival rate 9, 10. Immunotherapies have been developed using specific antibodies, PD-1-PD-L1 checkpoint inhibitors, and improved antitumor immune responses to block PD-1-PD-L1 interactions and restore immune cell surveillance of tumor cells 11. Although the development of targeted immune therapies has improved clinical outcomes, patient survival rates remain low due to the occurrence of immune resistance 12-14. Understanding the molecular mechanisms responsible for stabilization and upregulation of PD-L1 protein expression in CRC is critical. Thus, identifying the inhibitory regulation of PD-L1 protein abundance by the ubiquitin proteasomal system is necessary for developing a new therapeutic approach to mitigate PD-L1-driven tumorigenesis.

Post-translational modification (PTM) is the covalent alteration of proteins subsequent to protein biosynthesis. PTMs are essential for metabolism, cellular growth, and other processes. Among several PTMs, ubiquitination is a process in which ubiquitin moieties attach to target substrates, signal for proteasomal degradation, and regulate several cellular processes 15. On the contrary, deubiquitinating enzymes (DUBs) facilitate the removal of ubiquitin moieties from specific ubiquitin-conjugated substrates to modulate their functions. DUBs regulate several cellular processes such as cell cycle, cell growth, apoptosis, and signal transduction 16, 17. The human genome includes approximately 100 DUBs that can be classified into seven different families 18. Among them ubiquitin-specific proteases (USPs) is the largest and well-studied subfamily 19. Several reports suggest that changes in the expression of USPs are closely associated with the progression of various human cancers 20-22. However, the expression levels of USPs in patients with CRC, and the clinical correlation with tumorigenesis, have yet to be thoroughly investigated.

Understanding the cause of elevated PD-L1 expression in TME and its prognostic implications in CRC at the molecular level demands advanced analytical methodologies, including single-cell RNA sequencing (scRNA-seq). Combining scRNA-seq analysis with deubiquitylation pathway studies enables an in-depth analysis of DUB genes in individual cells, uncovering cell-specific gene expression patterns and DUB-mediated regulatory mechanisms that elucidate the complex cellular interactions within the TME. This study aims to investigate the expression of DUBs and their roles in PD-L1-mediated CRC pathogenesis and progression to establish novel therapeutic targets and strategies.

This is the first study to use high-dimensional scRNA-seq analysis to examine the effects of genome-wide USPs in heterogeneous leukocytes of CRC. The findings indicate the presence of unique USPs in cancer, particularly within cancer immune cells, implying their potential to serve as immunotherapy targets. We observed the involvement of PD-L1 expression in particular types of immune cells associated with CRC, including T-cells, macrophages, classical monocytes, and dendritic cells, with a proportional increase seen in the expression of cancer progression-specific USPs, including USP10, USP14, USP18, USP32, USP33, and USP39. Our in-depth scRNA-seq analysis revealed a significant correlation between the expression of PD-L1 and ubiquitin-specific protease 32 (USP32) within the immune cell types from human and mouse colon tumor tissues. Consequently, through wet lab experiments we validated that USP32 functions as a protein stabilizer of PD-L1 in CRC. We also demonstrated that USP32 binds with, stabilizes, deubiquitinates, and prolongs PD-L1 half-life by preventing protein degradation. To strengthen the immunotherapeutic approach, we knocked out the *USP32* in CRC cells, resulting in reduced cell proliferation, migration, invasion, and colony formation through a reduction in PD-L1 protein levels. Finally, we demonstrated that the loss of *USP32* attenuated PD-L1-driven colorectal tumorigenesis in a mouse xenograft model, underscoring its therapeutic potential.

## Results

### Cell-type clustering and annotation of human scRNA-seq samples

**Figure 1:**
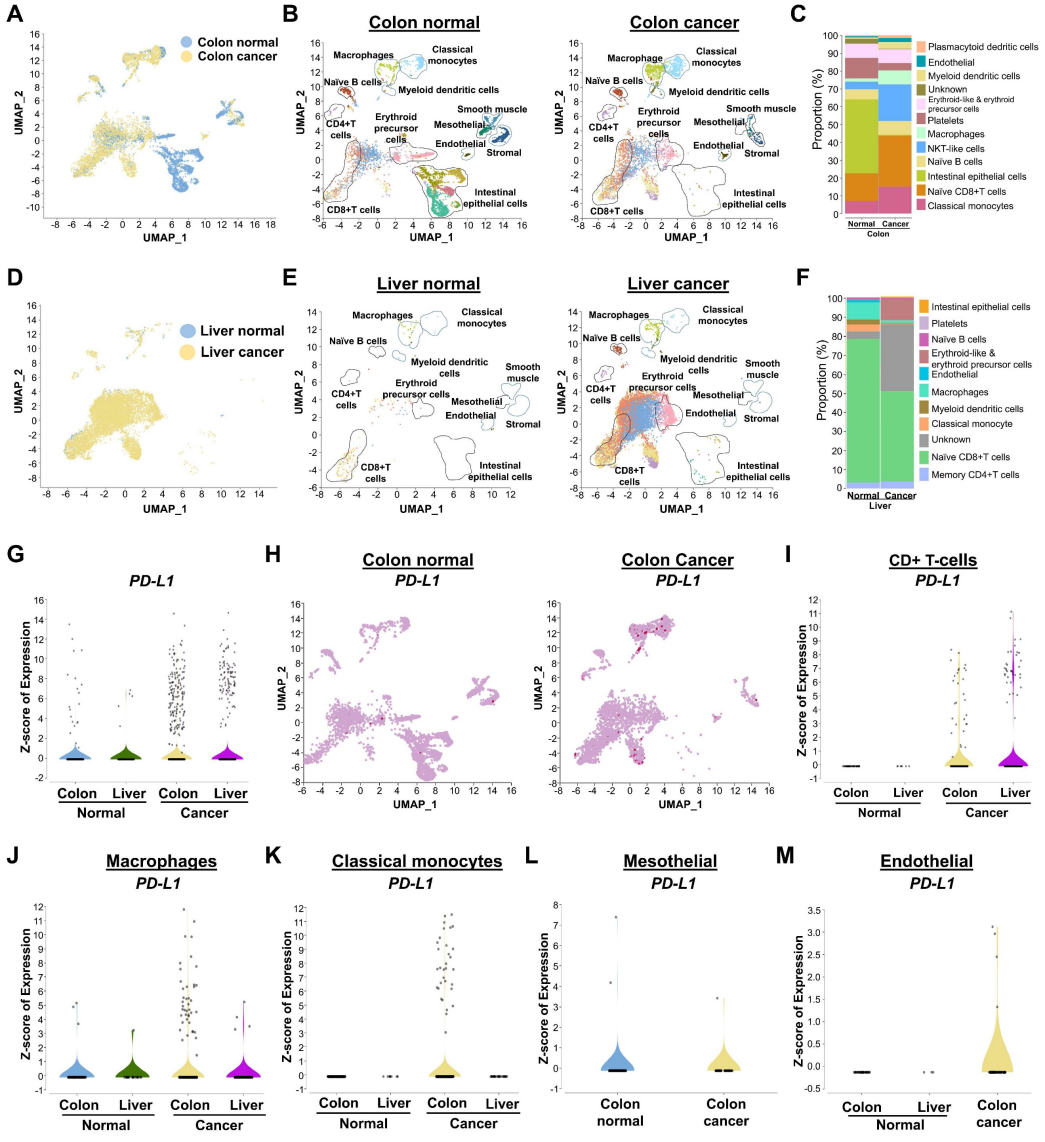
** The identification of heterogeneous immune cell populations and gene expression differences between colon or liver tumor tissues and their respective host tissue counterparts by human scRNA-seq analysis. (A)** UMAP visualization of heterogeneous clusters and differences between the colon tissue and colon cancer tissue groups. **(B)** Different cell types from colon tissue and colon cancer tissue groups were identified by the expression of reference marker genes and visualized using UMAP. **(C)** Population differences between the two groups from identified cells types. **(D)** Heterogeneous clusters differentiation from the liver tissue and liver cancer tissue groups visualized in UMAP. **(E)** Cell types identification of different clusters from liver tissues by the reference marker gene expression**. (F)** Comparison of cell type population between liver tissue and liver cancer tissue groups. (**G**) Comparison of *PD-L1* gene expression in total cell population between colon or liver normal tissues and colon or liver cancer tissues. (**H**) Overall expression of *PD-L1* in different cell types between the colon tissue and colon cancer tissue groups demonstrated in UMAP. (**I-M**) Violin plot for comparisons of *PD-L1* level analysis expression in cancer tissues from colon or liver and its control tissue in specific immune cell types (I) CD+ T-cells, (J) Macrophages, (K) Classical monocytes, (L) Mesothelial, (M) Endothelial.

This study compares cancer-specific expression of PD-L1 and associated DUBs during progression of colon and liver cancer. We used 26 scRNA-seq samples and pre-processed all samples to create a well-integrated cell population, using the Uniform Manifold Approximation and Projection (UMAP) program to analyze differences in cell populations between colon or liver cancer and their corresponding controls (Figures 1A and 1D). We applied shared nearest neighbor clustering to differentiate colon or liver cancer cells from host control cells, and heterogeneous cell clusters were identified using cell annotation analysis based on expression of their marker genes (Figures 1B and 1E). These clusters consisted of major immune cell types, such as naïve B cells, T-cells, macrophages, classical monocytes, myeloid dendritic cells, erythroid cells, and endothelial cells (Figures 1B and 1E). The population disparities of identified cell types from colon or liver cancer exhibited greater numbers of specific cell types compared with the control group, including T-cells, macrophages, classical monocytes, and myeloid dendritic cells (Figure 1C and 1F). However, population disparities in liver cancer were less pronounced than those in colon cancer (Figures 1C and 1F). A heat map was used to illustrate the differently expressed genes (DEGs) in the major cell types (Figures S1-2).

### Distinguishing between *PD-L1* expression in human colon and liver cancer

An examination of *PD-L1* expression indicated that colon cancer cohorts exhibited comparatively elevated levels in contrast to those of liver cancer cohorts (Figure 1G). Expression of *PD-L1* was significantly higher in colon and liver cancer compared with the corresponding control group (Figure 1H and Figure S3). Compared with liver cancer cell types, expression of *PD-L1* was relatively high in several colon cancer cell types, such as T-cells, macrophages, classical monocytes, and myeloid dendritic cells (Figure 1I-K, Figure S4). Interestingly, *PD-L1* was specifically shown to be highly expressed in mesothelial and endothelial colon cancer, but not in liver cancer cells (Figure 1L-M). Therefore, for further research, we concentrated on *PD-L1* expression and related DUBs in colon cancer.

### Genome-wide identification and expression analysis of USP gene family in human colon cancer

**Figure 2:**
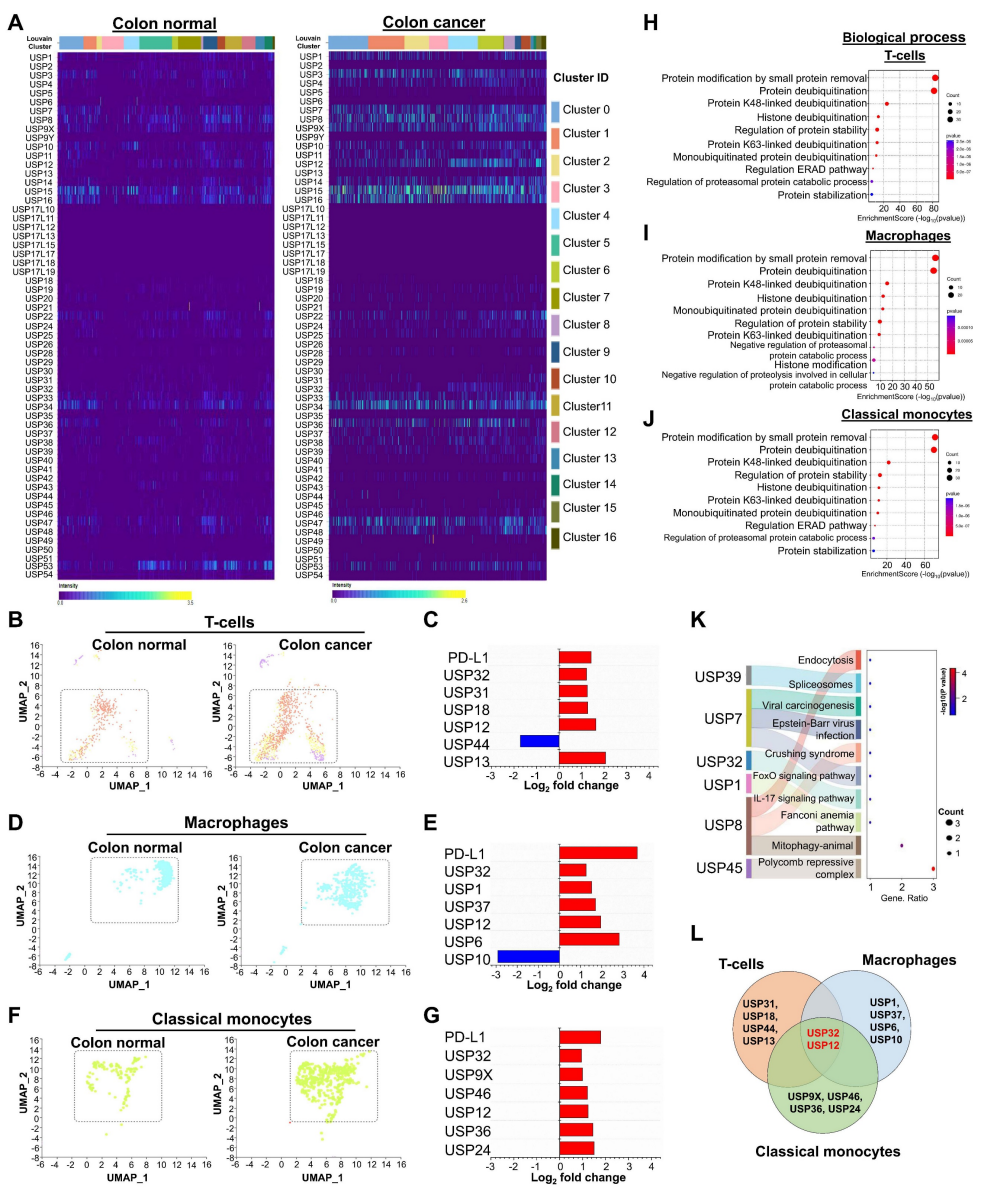
** scRNA-seq analysis of the entire USP family between colon normal tissue and colon cancer tissue groups. (A)** A heat map expression analysis was used to analyze genome-wide USP family genes from all cell types between colon tissue and colon cancer tissue groups. **(B-C)** Population differences of T-cells and the differentially expressed USP genes between the colon tissue and colon cancer tissue groups. **(D-E)** Population differences of macrophages and the differentially expressed USPs between the colon tissue and colon cancer tissue groups. **(F-G)** Disparities in classical monocytes populations and differently expressed USPs were discovered. **(H-J)** Gene ontology performance particularly, biological process for (H) T-cells, (I) macrophages and (J) classical monocytes were performed and visualized. **(K)** The snaky and dot blot visualization predicted molecular pathways for the differentially expressed USPs from T-cells. **(L)** Venn diagram showed the comparison of differently expressed USPs between the T-cells, macrophages, and classical monocytes cell types.

For the first time, an entire set of USP gene families were selected from the Genecard database and the genome-wide expression of USP genes in colon cancer was examined using a heatmap. Expression of a number of USP genes differed markedly between the cancer and control groups (Figure 2A). To better understand the significance of USP expression in relation to PD-L1-linked prognosis in CRC, we concentrated on those USP genes that were altered in T-cells, macrophages, and classical monocyte cell types, as *PD-L1* expression was higher in these cell types (Figure 2B-G). Among various USPs, *USP32, USP31, USP18, USP12,* and* USP13* are markedly upregulated in T-cells (Figure 2C). *USP32, USP1, USP37, USP12*, and USP6 are elevated in macrophages (Figure 2E), while *USP32, USP9X, USP46, USP12, USP36,* and *USP24* are upregulated in classical monocytes of colon cancer relative to the control group (Figure 2G). According to the gene ontology (GO), the biological process of differentially expressed USPs in T-cells, macrophages, and classical monocytes were involved primarily in protein modification, deubiquitination by lysine (K) 48-linked deubiquitination, protein stability and protease activity (Figure 2H-J). These USPs participated in the functions of the peptidase complex and the cytoplasmic ubiquitin ligase complex within cellular components (Figure S5A-C), while the molecular function indicated that they were engaged in deubiquitinase activity, cysteine-type peptidase activity, and K48-specific deubiquitinase activity (Figure S5D-F,). Furthermore, these USP genes mostly govern immune-related processes (Figure 2K, Figure S6). Interestingly, *USP32* and *USP12* have been repeatedly found at elevated levels in T-cells, macrophages, and classical monocytes associated with colon cancer compared with levels in the control group (Figure 2L).

### Cell type clustering and annotation of mouse scRNA-seq samples

**Figure 3:**
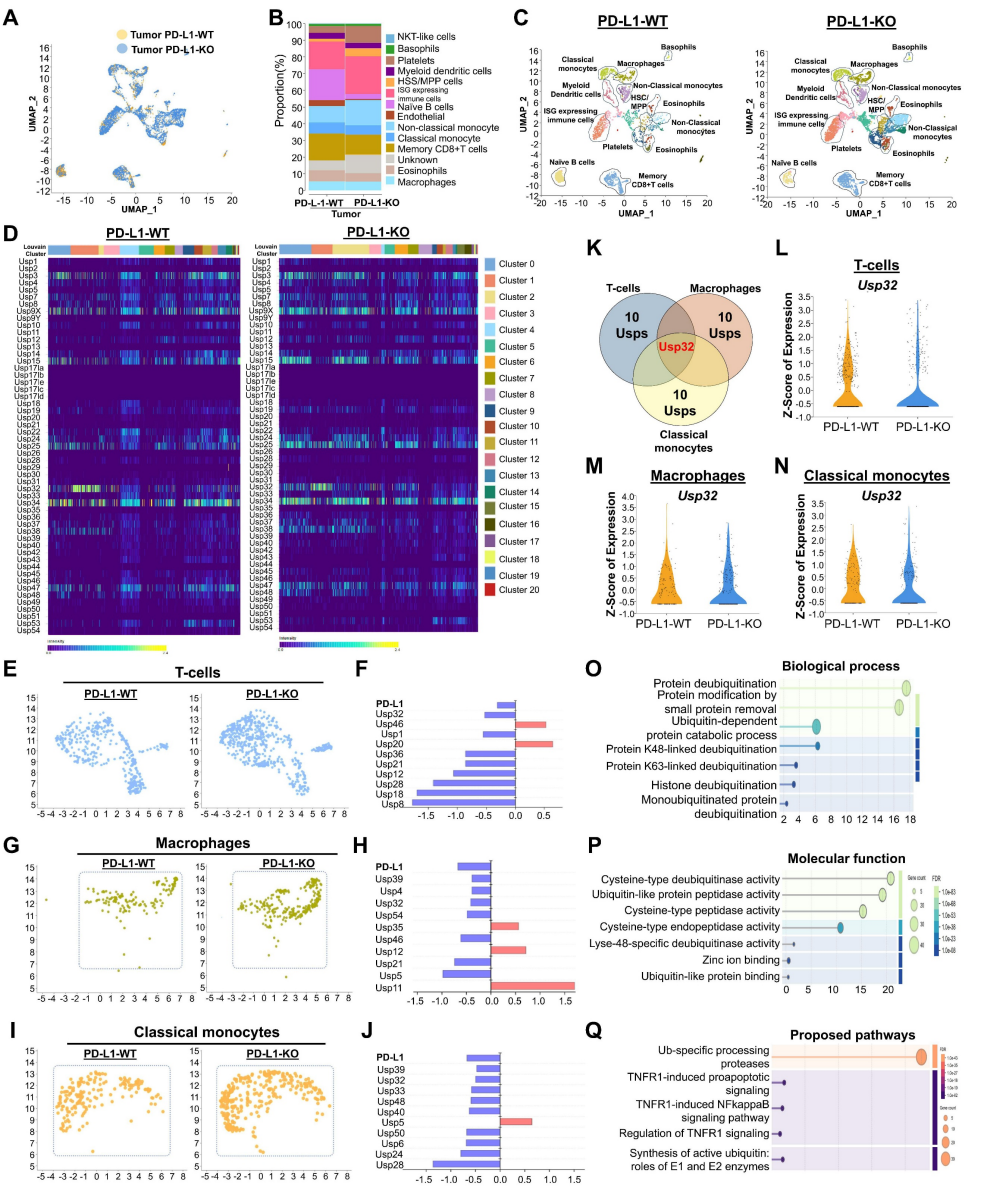
** The expression of the entire USP family in mouse colorectal cancer encoding a PD-L1 wild type and PD-L1 knockout scRNA-seq database. (A)** Visualization of heterogeneous immune clusters from PD-L1 wild type (PD-L1-WT) and PD-L1 knockout (PD-L1-KO) in mouse colorectal cancer. **(B)** Comparison of identified immune cell populations between the mice group were shown**. (C)** Cell type annotations revealed identification of heterogeneous immune cells from PD-L1-WT and PD-L1-KO mice groups**. (D)** Heat map expressions of genome-wide USPs from different cell clusters were visualized and compared between the mice group**. (E-F)** Population differences of T-cells and the differentially expressed USP genes between the PD-L1-WT and PD-L1-KO mice groups.** (G-H)** Population differences and the differentially expressed USPs of macrophages from PD-L1-WT and PD-L1-KO mice groups were shown. **(I-J)** Population differences and the differentially expressed USPs of classical monocytes from PD-L1-WT and PD-L1-KO mice groups were shown. **(K)** The differences in differentially expressed USPs from T-cells, macrophages and classical monocytes were compared by Venn diagram. **(L-N)** Violin plot showed comparison of *Usp32* expression between the mice groups obtained from (L) T-cells, (M) macrophages, and (N) classical monocytes cell types. **(O-Q)** The functional annotation of differentially expressed USPs from T-cell type were performed and visualized (O) biochemical process (P) molecular function, (Q) proposed molecular pathways.

In order to gain more insight into the relationship between USPs and PD-L1-driven immune cell trafficking in TME, we then analyzed the genome-wide expression of USP genes in the presence or absence of PD-L1 in mouse CRC. To this end, we performed scRNA-seq analysis in a PD-L1 knockout (PD-L1-KO) colon cancer mice compared with PD-L1 wild type (PD-L1-WT) colon cancer mice group. The UMAP analysis from both groups of mice showed major cell types, including classical monocytes, macrophages, basophils, myeloid dendritic cells, non-classical monocytes, immune cells that express interferon-stimulated genes, eosinophils, B-cells, and T-cells (Figure 3A-C). Additionally, we analyzed the types of cell populations between PD-L1-WT and PD-L1-KO mice and identified significant disparities in cell type expression (Figure 3B-C, Figure S7), which elucidates the specific function of PD-L1 in the prognosis of CRC. Strongly expressed genes from diverse cell types were compared between two groups of mice and illustrated in a heat map (Figure S8,).

### Distinguishing USP genes expression in mouse T-cells, macrophages and classical monocytes

Next, we conducted an in-depth analysis of the interaction patterns among the entire USPs family of genes (Figure S9,) and their expression in relation to *PD-L1* expression in both PD-L1-WT and PD-L1-KO mice to determine their specific functions in CRC progression. The heat map results indicate differentially expressed USP genes in heterogeneous immune cells between both mouse groups (Figure 3D). To discern cell-specific changes of USPs and investigate the differences between PD-L1-WT and PD-L1-KO colon cancer mice, we analyzed T-cells, macrophages, and classical monocytes individually. In comparison with PD-L1-WT mice, the population of T-cells, macrophages, and classical monocytes was significantly elevated in PD-L1-KO mice (Figure 3E-J), indicating an enhancement of immune cells to counteract cancer.

Notably, expression of several USPs exhibited contrasting results, demonstrating downregulation in T-cells, macrophages, and classical monocytes cell populations when comparing two mice groups (Figure 3F, 3H and 3J), suggesting a correlation between these USPs and PD-L1 expression. Out of all the putative USP genes, *Usp32* alone emerged as a potential candidate that was downregulated in the populations of T-cells, macrophages, and classical monocytes (Figure 3K). This was consistent with our earlier findings from human scRNA-seq analysis (Figure 2). We also examined the expression level of *Usp32* in the presence and absence of *PD-L1* in the T cell, macrophages, and classical monocyte cell populations in the two groups of mice. It is evident that the PD-L1-WT mice showed clearly elevated *Usp32* expression, whereas PD-L1-KO mice had lower *Usp32* expression (Figure 3L-N, Figure S10). According to the GO of the differentially expressed USPs in T-cells, the biological process, cellular component, and molecular functions were primarily involved in protein modification and deubiquitination, specifically by K48-linked deubiquitination, and protease activity (Figure 3O-P, Figure S11A,). Additionally, they were involved in the ubiquitin-specific processing proteases pathway (Figure 3Q, Figure S11B,).

### Screening for putative DUBs identifies USP32 as a protein stabilizer of PD-L1

**Figure 4:**
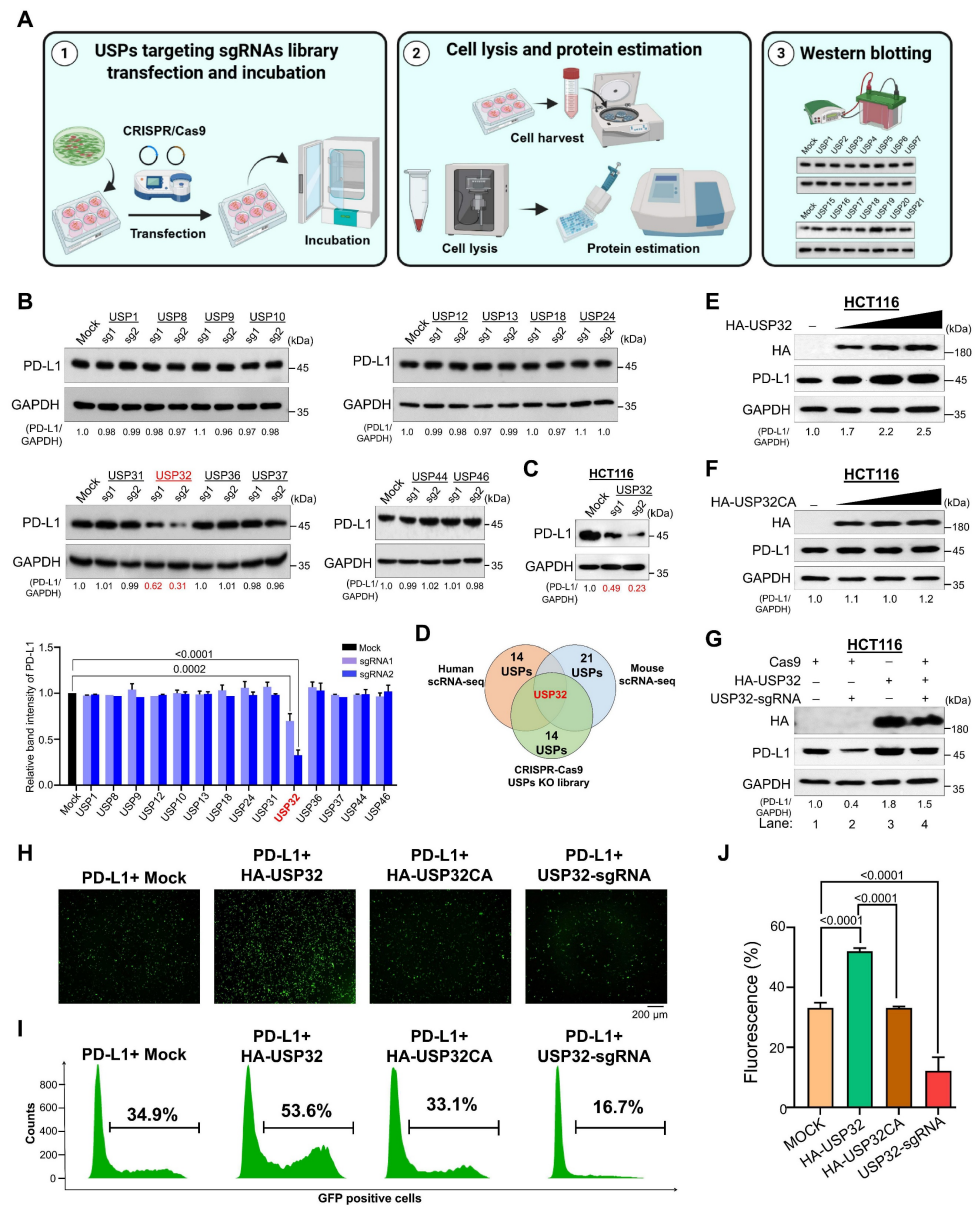
** Screening for USPs using CRISPR/Cas9-based sgRNA library targeting USP family that regulate PD-L1 protein levels. (A)** An overview of CRISPR/Cas9-sgRNA library preparation and identification for putative USPs that regulate PD-L1 protein expression by western blotting. **(B)** The sgRNAs with Cas9 transfected into HCT116 cells targeting DUB genes were used to estimate PD-L1 protein expression. Cas9 and scrambled sgRNA transfected cells were considered as mock controls. **(C)** The sgRNA targeting *USP32* on PD-L1 protein expression was analyzed in HCT116 cells. **(D)** Venn diagram showed the comparison of differently expressed USPs obtained from CRISPR/Cas9-based screening, human scRNA-seq, and mouse scRNA-seq analyses. **(E)** An increasing concentration of HA-USP32 or **(F)** HA-USP32CA were transfected to assess PD-L1 expression. **(G)** HA-USP32 was reconstituted to analyze PD-L1 expression in USP32-silenced cells. The band intensity of PD-L1 protein was quantified using ImageJ tool and mentioned below the blot (PD-L1/GAPDH). **(H)** Representative microscopic images of GFP-PD-L1-expressing cells transfected with the indicated constructs. **(I)** Histograms from FACS analysis. GFP-tagged PD-L1 was transfected along with the indicated constructs. Cells within the gated region are shown, and the number indicates the proportion of GFP-positive cells. **(J)** The percentage of GFP-positive cells representing PD-L1 was plotted and *P* values are indicated on the figures.

Next, we wished to knockdown the putative DUB genes derived from scRNA-seq data in order to examine the effect of these putative DUBs on PD-L1 expression at the post-translational level. To this end, we used our previously established DUB knockout library consists of single guide RNAs (sgRNAs) targeting USP family genes (Figure 4A) 23-25. Our secondary screening revealed that the knockdown of USP32 resulted in reduced PD-L1 protein expression by western blot (Figure 4B). This observation was cross-confirmed in colon cancer cell line HCT116 cells (Figure 4C, Figure S12A). Thus, we identified USP32 as a potential DUB candidate obtained from human scRNA-seq, mouse scRNA-seq and CRISPR/Cas9-based screening for putative USPs regulating PD-L1 protein expression (Figure 4D). To further investigate the effect of USP32 on PD-L1, we transfected increasing concentration of USP32 in HCT116 and HEK293 cells and analyzed PD-L1 protein levels. The endogenous and exogenous PD-L1 protein level was gradually increased when USP32 was increased in a concentration dependent manner (Figure 4E, Figure S12B and S13A). However, USP32 catalytic mutant showed no upregulation effect on PD-L1 protein levels (Figure 4F, Figure S12C and S13B). The sgRNA2 targeting *USP32* showed a reduced PD-L1 protein expression (Figure 4G, lane 2, Figure S12D and S13C), while reconstitution of USP32 in USP32-depleted cells regained the PD-L1 expression (Figure 4G, lane 2 vs 4, Figure S12D and S13C). Overexpression of USP32 augmented intracellular intensities of green fluorescent protein (GFP)-tagged PD-L1, whereas the catalytic mutant USP32 did not exhibit this effect (Figure 4H-J). In contrast, the knockdown of USP32 diminished GFP fluorescence intensities (Figure 4H-J), suggesting that USP32 functions as a specific deubiquitinase for the PD-L1 protein.

### The correlation between *USP32* and *PD-L1* in human colon cancer

**Figure 5:**
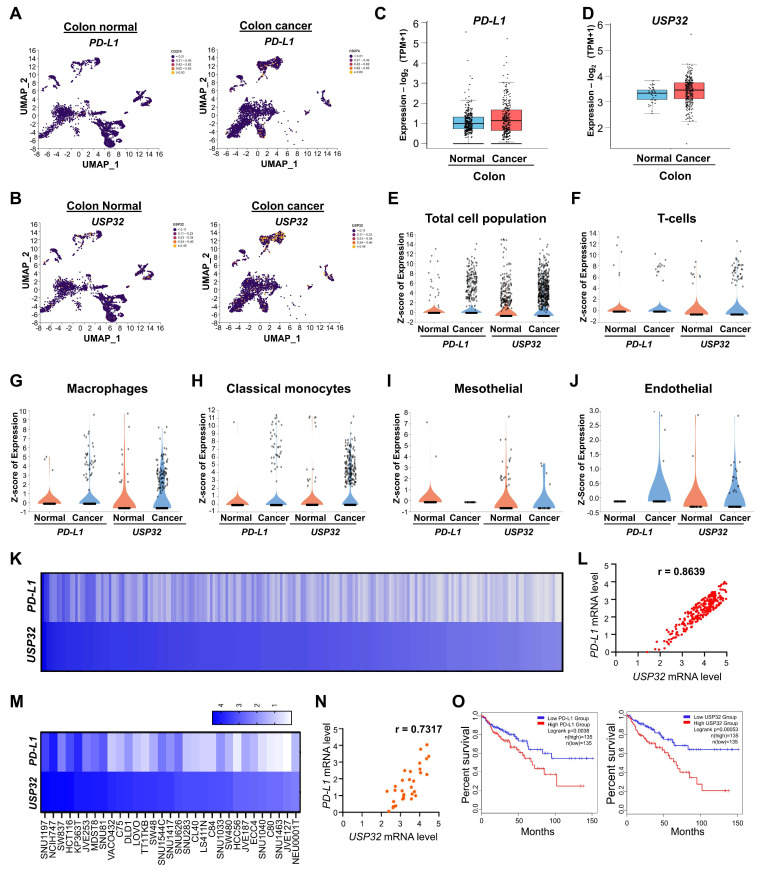
** Clinical correlation between *USP32* and *PD-L1* mRNA levels in colorectal cancers. (A-E)** The *USP32* and *PD-L1* expression was analyzed from scRNA-seq data set obtained from human colon cancer samples relative to its control tissues and from (C-D) TCGA data set. The correlation between *USP32* and *PD-L1* particularly from **(F)** T-cells, **(G)** macrophages, **(H)** classical monocytes, **(I)** mesothelial, and **(J)** endothelial. **(K-L)** *USP32* and *PD-L1* expression by heat map across several cancer cell lines are derived from CCLE database. Expression levels of *USP32* from high to low with corresponding *PD-L1* values. **(M)** *USP32* and *PD-L1* expression in various colon cancer cell lines. **(N)** The correlation between *USP32* and *PD-L1* mRNA expressions with Pearson correlations (r) value. **(O)** The overall survival probability of groups expressing low *USP32* expression level (n = 135) and high *USP32* expression level (n = 135) from TCGA database were analyzed using GEPIA 2.

We analyzed the correlation between the expression of *USP32* and *PD-L1* in colon cancer compared to the control group. Like *PD-L1*, *USP32* is more highly expressed in colon cancer than control group (Figure 5A-E), particularly elevated expression was observed in specific cell types including T-cells, macrophages, classical monocytes, mesothelial, and endothelial cells (Figure 5F-J). The correlation between *USP32* and *PD-L1* expression across wide panel of cancer cells using the CCLE database showed that the high score for *USP32* mRNA level was proportional to the *PD-L1* mRNA level with r value 0.8639 (Figure 5K-L, Table S5). Particularly showing positive correlation between *USP32* and PD-L1 in colon cancer cell lines with a r value of 0.7317 (Figure 5M-N). Moreover, Kaplan-Meier analysis of TCGA survival data indicated that elevated expression of *USP32* and *PD-L1* correlates with worse survival in colon cancer patients (Figure 5O), indicating that high *USP32* expression may be associated with an adverse prognosis in colon cancers.

### Loss of *USP32* suppresses CRC growth *in vitro*

**Figure 6:**
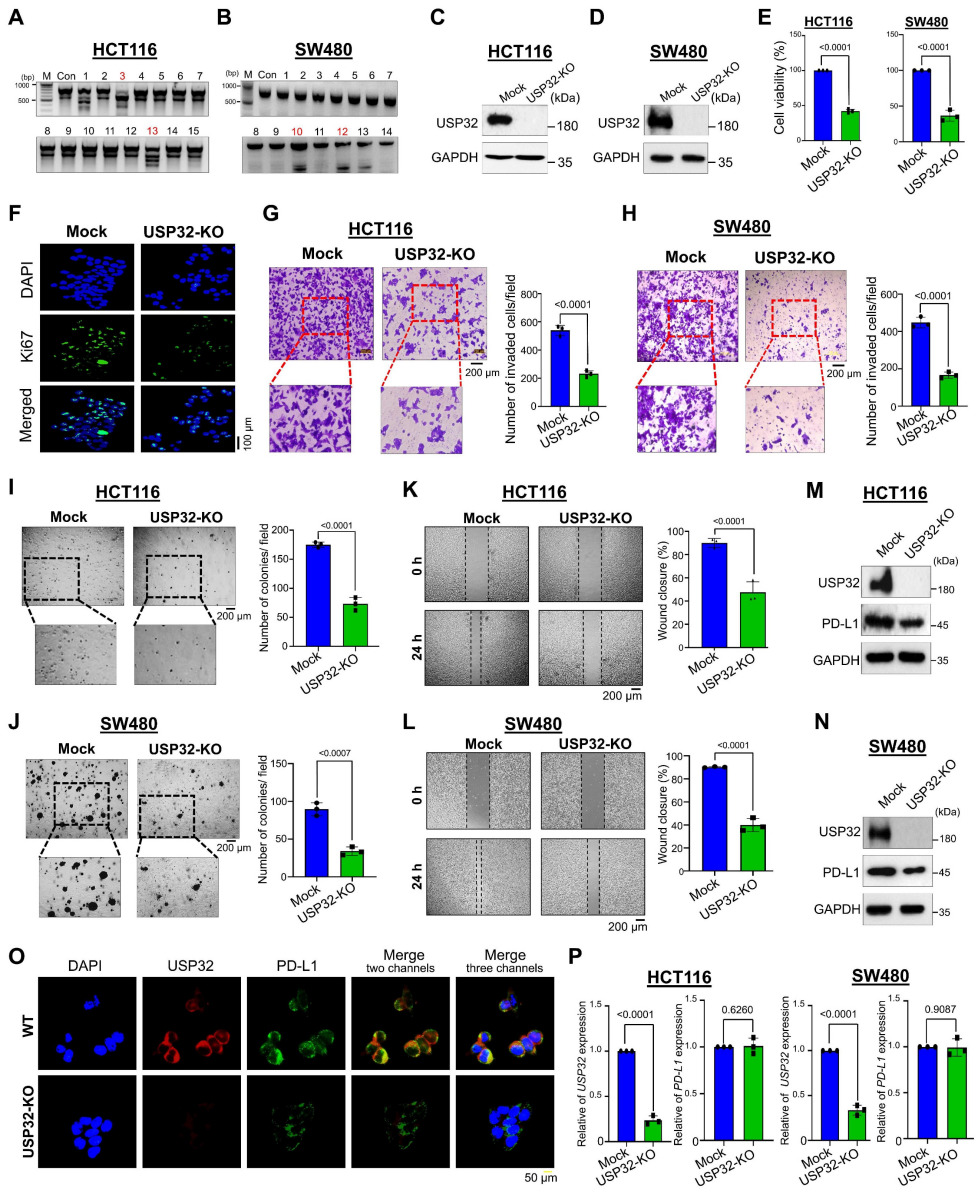
** Loss of *USP32* inhibits carcinogenic activity in HCT116 and SW480. (A-B)** *USP32* gene knockout clones were screened by a T7E1 assay in (A) HCT116 and (B) SW480 cells. **(C-D)** Endogenous USP32 expression was evaluated in (C) USP32-KO HCT116 cells and (D) USP32-KO SW480 cells by western blotting. **(E-L)** The impact of USP32-depletion in HCT116 and SW480 cells was analyzed by (E) cell viability using CCK-8 kit (F) cell proliferation by immunofluorescence staining with proliferative marker Ki67 (green) in HCT116 cells, Scale bar = 100 µm, (G) cell invasion by transwell cell-invasion assay in HCT116 cells and (H) SW480 cells, (I) colony formation by soft agar assay in HCT116 cells and (J) SW480 cells, Scale bar = 200 µm, (K) cell migration by wound-healing assay in HCT116 cells and (L) SW480 cells, Scale bar = 200 µm. **(M-N)** The effect of USP32-depletion on PD-L1 expression was analyzed by western blotting in (M) HCT116 cells and (N) SW480 cells, and **(O)** immunofluorescence staining with specific antibodies in HCT116 cells. **(P)** The mRNA expression of *USP32* and *PD-L1* by qRT PCR. GAPDH was used as a control.

Because scRNA-seq analysis revealed that *USP3*2 expression was elevated in colon cancer, we hypothesized that knockout of the *USP32* gene could impede cell proliferation and colon cancer growth *in vitro*. The two sets of sgRNAs targeting the *USP32* gene (exon 3) were designed (Figure S14A). The sgRNA2 targeting the *USP32* showed high cleavage efficiency than sgRNA1 (Figure S14B), which is in line with its efficiency in reducing USP32 protein expression (Figure S14C). Thus, we transfected sgRNA2 along with RFP-expressing Cas9 to knockout the* USP32* gene in HCT116 and SW480 cells. The RFP-expressing cells were sorted and subjected to single cell dilution and then seeded onto a 96 well plate for expansion (Figure S14D). The clones were subjected to a T7E1 assay for screening *USP32* knockout clones. The *USP32* knockout clone #3 and clone #10 of HCT116 and SW480 respectively (Figure 6A-B), showing cleavage efficiency and complete aberration of USP32 protein expression was selected (Figure 6C-D). The gene disruption in *USP32* knockout clone of HCT116 and SW480 cells (hereafter USP32-KO) were confirmed by Sanger sequencing for further functional analysis (Figure S14E-F). Next, we sought to determine the role of USP32 on cell viability and proliferation in both USP32-KO HCT116 and SW480 cells. The results showed a significant reduction in cell viability (Figure 6E), proliferation (Figure 6F), invasion (Figure 6G-H), colony formation (Figure 6I-J) and migration (Figure 6K-L) in USP32-KO HCT116 and SW480 cells when compared with mock controls, suggesting that the loss of *USP32* inhibits CRC growth.

### USP32 binds with, deubiquitinates, and prolongs PD-L1 protein half-life

Next, we demonstrated that USP32-KO cells exhibit reduced PD-L1 protein as evident by immunoblotting (Figure 6M-N) and immunofluorescence assay (Figure 6O). Moreover, the mRNA level of USP32 was completely abolished in USP32-KO cells, while the mRNA level of PD-L1 was not altered in USP32-KO cells compared with wild type HCT116 and SW480 cells (Figure 6P), indicating that USP32 regulates PD-L1 at a post-translational level.

**Figure 7:**
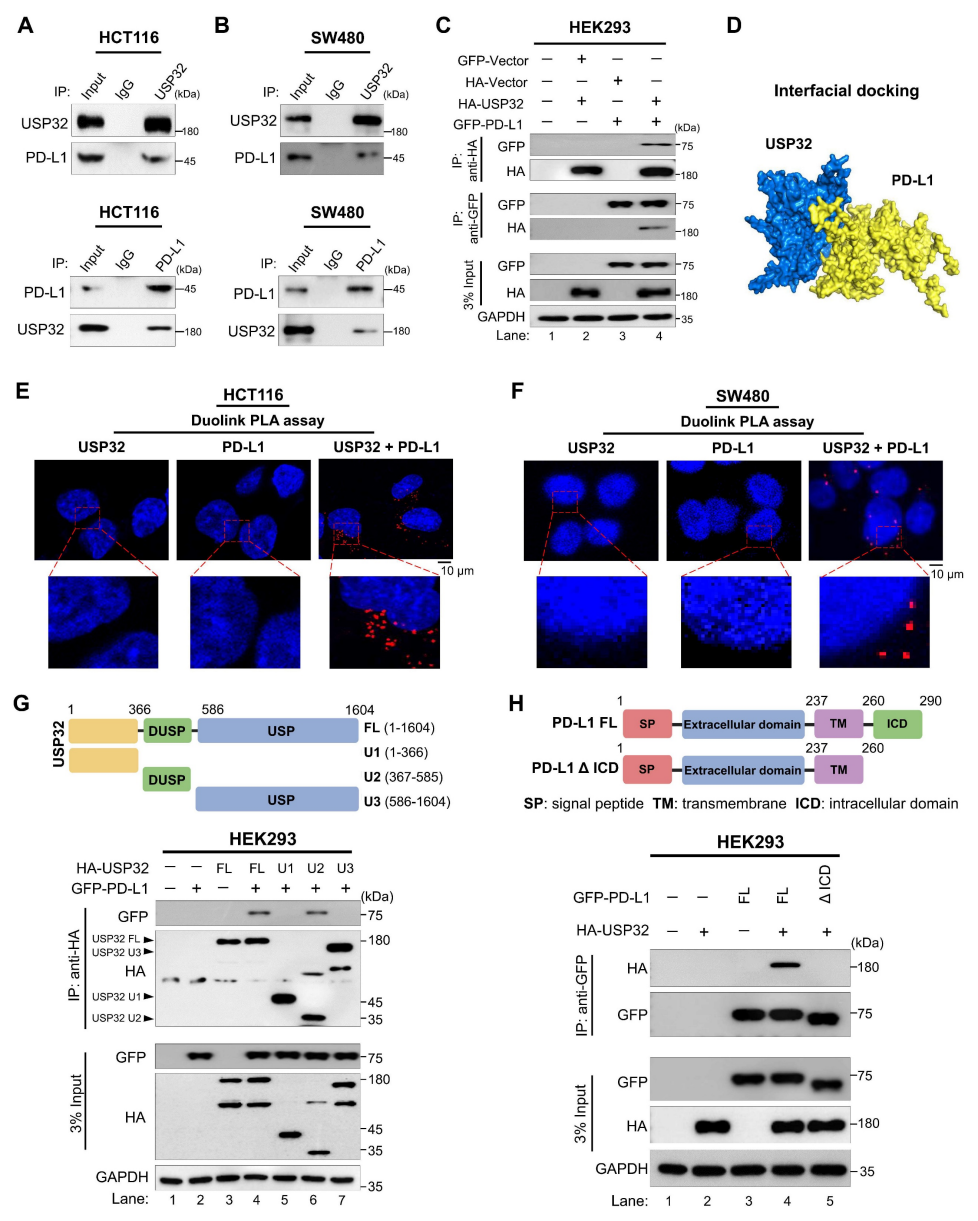
** USP32 interacts with PD-L1 protein. (A-B)** Interactions between USP32 and PD-L1 proteins in (A) HCT116 and (B) SW480 cells using specific antibodies and **(C)** ectopic HA-USP32 and GFP-PD-L1 proteins in HEK293 cells using tagged antibodies were examined by immunoprecipitation followed by western blotting. **(D)** Interfacial protein-protein docking score predicted between USP32 and PD-L1 proteins. **(E-F)** The interaction between endogenous USP32 and PD-L1 proteins using specific antibodies in (E) HCT116 and (F) SW480 cells. Scale bar: 10 μm. **(G)** Schematic representation of USP32 truncations (upper panel) and co-immunoprecipitation assay to investigate the interaction between USP32 truncations with full length PD-L1. **(H)** Schematic representation of PD-L1 truncations (upper panel) and co-immunoprecipitation assay to investigate the interaction between PD-L1 truncations with full length USP32.

To illustrate the mechanism of USP32-mediated stabilization of PD-L1 in colon cancer progression, we sought to investigate the physical association between USP32 and PD-L1 proteins. The immunoprecipitation assay using endogenous USP32 or PD-L1 antibodies and tagged antibodies showed that these two proteins interacted with each other endogenously (Figure 7A-B) and exogenously (Figure 7C). To support our results, the interaction between USP32 and PD-L1 showed a significant confidence score 0.8079 through an interfacial docking model using HDOCK 26 (Figure 7D). Duolink proximity ligation assay (PLA) assay revealed that USP32 interacts with PD-L1, PLA dots appeared when both USP32 and PD-L1 antibodies were used (Figure 7E-F). Next, we investigated the minimal specific regions of USP32 and PD-L1 that were critical for their interaction. We generated truncations of USP32, U1 (1-366), U2 (367-585) consists of N-terminal DUSP domain, and U3 (586-1604) consists of C-terminal USP domain (Figure 7G, upper panel). Interaction studies showed that the N-terminal truncation U2 in USP32 binds with PD-L1, suggesting that DUSP domain of USP32 is critical for USP32-PD-L1 interaction (Figure 7G, lane 6, lower panel). Conversely, we generated PD-L1-ΔICD (1- 260) truncation consists of N-terminal extracellular and a transmembrane domain but lacking C-terminal intracellular domain (ICD) (Figure 7H, upper panel). Binding studies showed that the PD-L1-ΔICD did not show any interaction with USP32, indicating that intracellular domain of PD-L1 contributes to the USP32-PD-L1 interaction (Figure 7H, lower panel).

**Figure 8:**
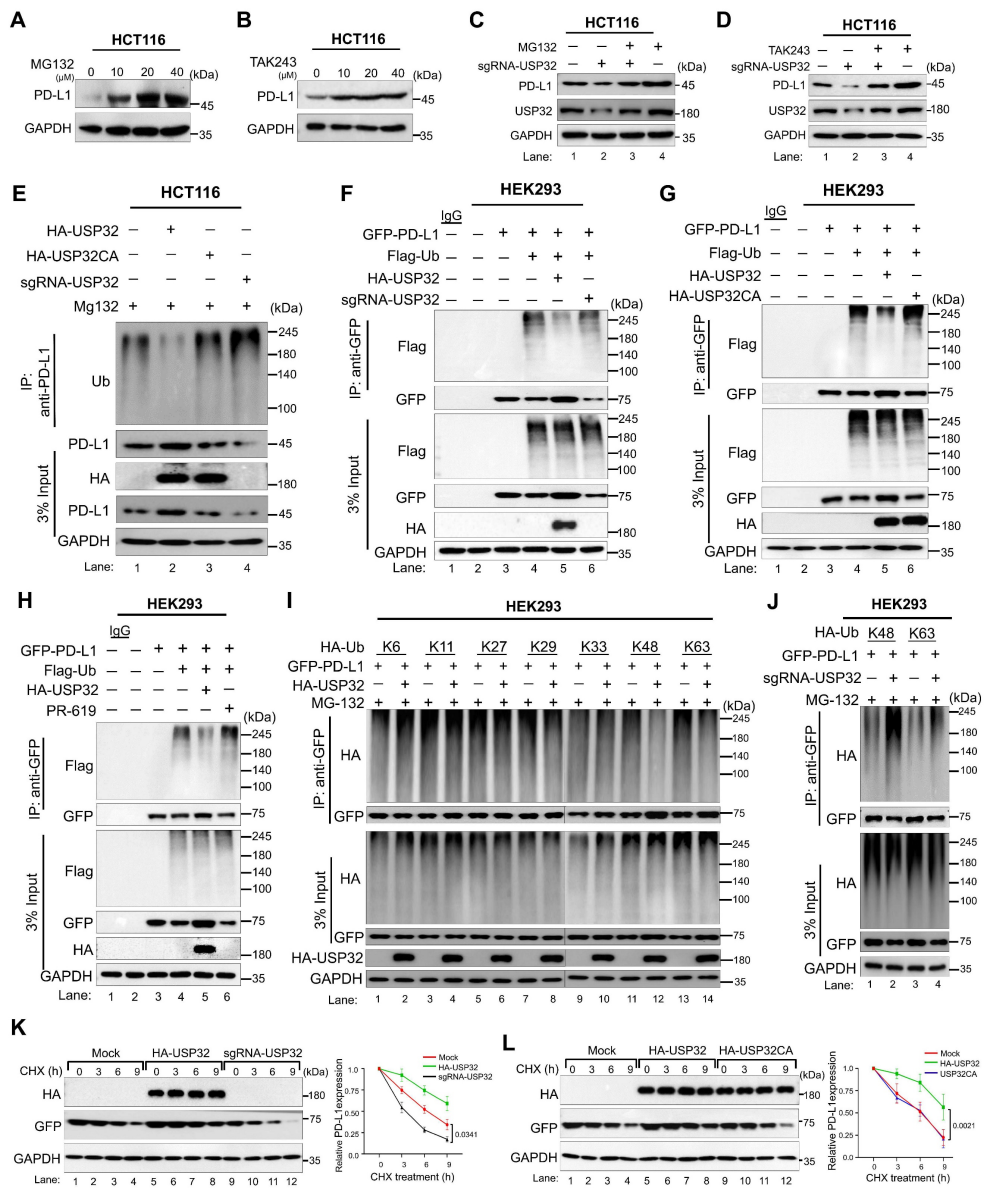
** USP32 deubiquitinates and prolongs the half-life of PD-L1 protein. (A-B)** HCT116 cells were subjected to the MG132 and TAK243 for 6 h. **(C-D)** The impact of USP32 depletion on PD-L1 level in the presence of (C) MG132 (20 µM) and (D) TAK243 (20 µM). **(E)** The endogenous PD-L1 polyubiquitination was analyzed in the presence of USP32, USP32CA, and sgRNA targeting USP32 in HCT116 cells. **(F-H)** The exogenous PD-L1 polyubiquitination in the presence of (F) USP32 and sgRNA targeting USP32, (G) USP32 and USP32CA, (H) USP32 and DUB inhibitor (PR-619) in HEK293 cells. **(I)** The deubiquitinating activity of USP32 on specific types of ubiquitin chains on PD-L1 protein. GFP-PD-L1 along with several HA-ubiquitin mutants were co-transfected into HEK293 cells followed by immunoprecipitation using anti-GFP antibody and immunoblotted with anti-HA antibody. WT is wild type ubiquitin; KR, Lysine is mutated to Arginine. **(J)** The effect of USP32 depletion on K-48 and K-63 linked ubiquitination of exogenous PD-L1 protein by immunoprecipitation in 293T cells. **(K-L)** The half-life of PD-L1 was estimated in the presence (K) HA-USP32 and sgRNA targeting *USP32*, and (L) HA-USP32 and HA-USP32CA in HEK293 cells by treating CHX (250 μg/mL).

The dose-dependent increase in MG132 and TAK243 led to the accumulation of PD-L1 protein level (Figure 8A-B, Figure S15A-B). Moreover, USP32-depletion-mediated reduction in PD-L1 protein level was also reversed by the treatment of MG132 or TAK243, suggesting that USP32 regulates PD-L1 protein level through proteasomal degradation (Figure 8C-D, Figure S15C-D). We therefore sought to determine the deubiquitinating activity of USP32 on PD-L1 protein. To this end, USP32 or USP32 catalytic mutant (USP32CA) was transfected and PD-L1 polyubiquitination was investigated. The PD-L1 polyubiquitination was reduced by overexpressing USP32 (Figure 8E, lane 2, Figure S16A), while no deubiquitinating activity was observed by USP32CA (Figure 8E, lane 3, Figure S16A). Similarly, USP32 showed deubiquitinating activity on ectopically expressed PD-L1 protein (Figure 8F-G, lane 5), while no deubiquitinating activity exhibited by USP32CA (Figure 8G, lane 6). In contrast, depletion of USP32 increased ubiquitin smear conjugated with PD-L1 when compared with mock control (Figure 8F, lane 6). Likewise, the DUB inhibitor (PR619) treated cells also showed high ubiquitin smear conjugated with PD-L1 (Figure 8H, lane 6), suggesting that deubiquitinating activity of USP32 prevents PD-L1 protein degradation. Furthermore, we sought to investigate the type of ubiquitin chains that form on PD-L1 protein and the impact of USP32 on those specific ubiquitination modifications. To this end, we co-transfected mutant ubiquitin constructs in which all the lysine residues were replaced by arginine residues retaining only one of the seven lysine sites (Lys (K)-6, K-11, K-27, K-29, K-33, K-48 and K-63). PD-L1 was ubiquitinated by all the types of ubiquitin linkages (Figure 8I). USP32 significantly removed K-48-linked polyubiquitin chains (Figure 8I, lane 12), while the knockdown of USP32 increased the K-48 linked polyubiquitination of PD-L1 (Figure 8J, lane 2, Figure S16B), suggesting that USP32 primarily deubiquitinates K-48-linked polyubiquitin chains from PD-L1 protein.

Next, we investigated the effect of USP32 on PD-L1 protein turnover. To this end, we used the protein synthesis inhibitor cycloheximide (CHX) and analyzed the expression of PD-L1 by overexpressing USP32. The PD-L1 half-life was significantly prolonged by the USP32 (Figure 8K-L, lane 5-8), while USP32-depletion led to a reduction in the PD-L1 half-life (Figure 8K, lane 9-12). However, the USP32CA did not extend PD-L1 half-life (Figure 8L, lane 9-12), indicating that deubiquitinating activity of USP32 prevented PD-L1 degradation and subsequently extended PD-L1 protein turnover.

### Loss of *USP32* inhibits PD-L1-mediated carcinogenesis *in vitro*

**Figure 9:**
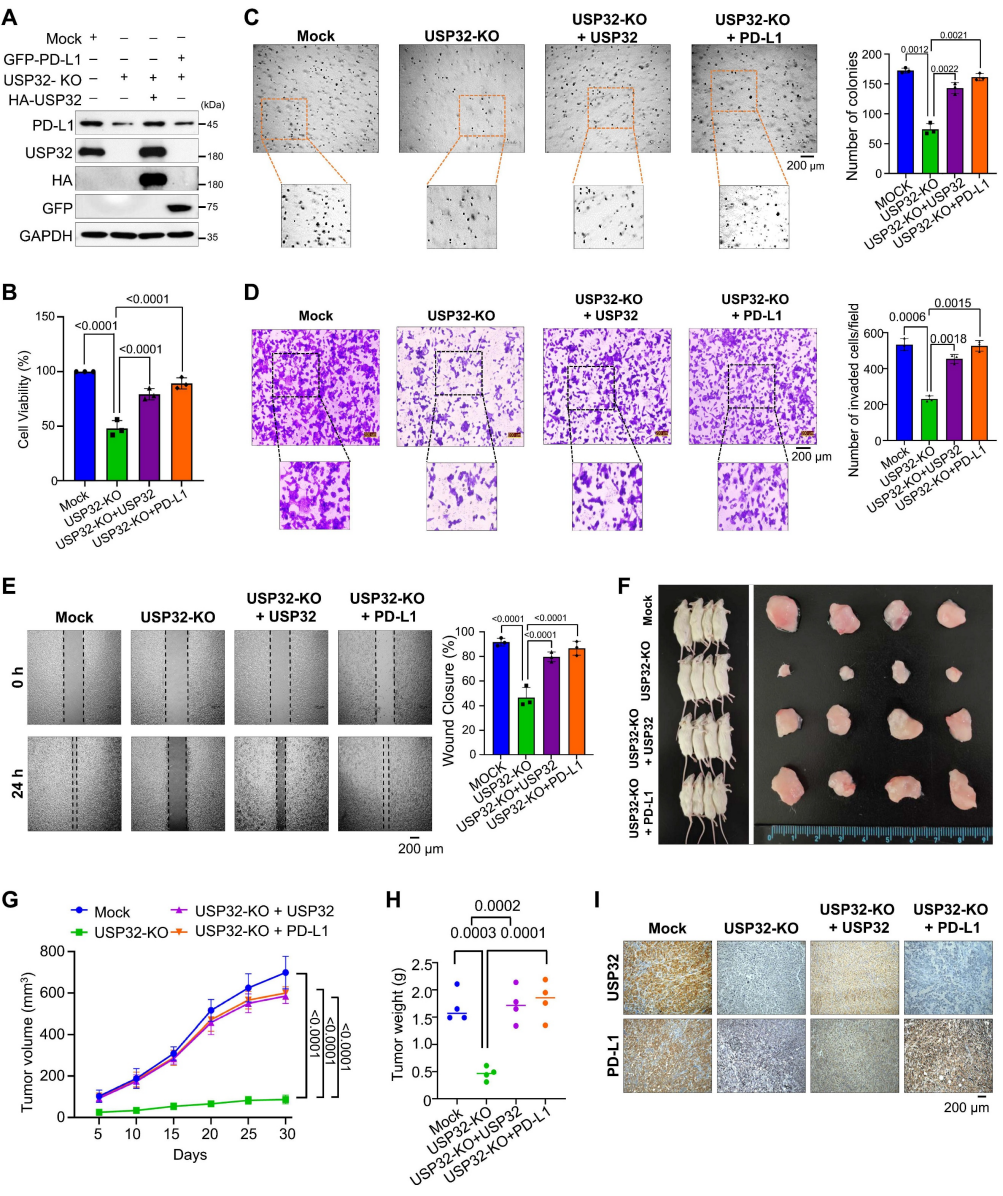
** The loss of *USP32* attenuates PD-L1-mediated colorectal tumorigenesis. (A)** The expression levels of USP32 and PD-L1 in Mock, USP32-KO and USP32-KO cells reconstituted with USP32 or PD-L1 were estimated. **(B-E)** The experimental groups were subjected for (B) cell viability (C) colony formation (D) invasion and (E) migration, Scale bar = 200 µm.** (F)** Xenograft models were established by subcutaneous injection of HCT116 cells mock, USP32-KO, and USP32-KO cells reconstituted with USP32 or PD-L1 into the right flanks of NSG mice. Tumors were surgically removed from mice at the end of the study (right panel). **(G)** Tumor volumes were recorded every five days over a 30-day period. **(H)** Tumor weights were measured following euthanasia and are presented graphically (n = 4 per group). **(I)** Immunohistochemical analysis was performed for mouse tumors tissues. Scale bar = 200 µm.

To investigate the stabilization effect of USP32 on PD-L1-driven carcinogenesis, we used a USP32-KO clones from HCT116 and SW480 cells showing low PD-L1 protein level and a USP32-KO clone reconstituted with either USP32 or PD-L1 for several functional evaluations (Figure 9A and Figure S17 and S18A). The USP32-KO HCT116 and SW480 cells showed reduced cell proliferation, while reconstitution with USP32 or PD-L1 regained the cell viability (Figure 9B and Figure S18B). The USP32-KO clones from HCT116 and SW480 cells had fewer colonies compared with the mock control in an anchorage-independent colony formation assay, whereas USP32-depleted cells reconstituted with USP32 or PD-L1 had a higher number of colonies (Figure 9C and Figure S18C). Likewise, a significant reduction was observed in cellular invasion (Figure 9D and Figure S18D) and migration (Figure 9E and Figure S18E) in USP32-KO clones, which was reversed when reconstituted with either USP32 or PD-L1 into USP32-KO clones, suggesting that the loss of USP32 prevents PD-L1-mediated oncogenic activity.

### USP32 promotes PD-L1-mediated tumor progression

To corroborate the USP32-mediated stabilization effect on PD-L1-driven oncogenic transformation *in vivo*, we subcutaneously injected USP32-KO, USP32-KO reconstituted with USP32 or PD-L1 along with mock cells into the right flanks of NOD scid γ (NSG) mice. The tumor volume and weight was reduced in the mice injected with USP32-KO (Figure 9F-H). In contrast, USP32-KO reconstitution with either USP32 or PD-L1 was associated with an increase in tumor volume and weight (Figures 9G-H). The mice xenograft tumor tissues exhibited low expression of PD-L1 in USP32-KO group, while reconstitution with either USP32 or PD-L1 restored PD-L1 expression (Figure 9I). Altogether, our results suggest that depletion of USP32 destabilizes PD-L1 protein and subsequently hampers PD-L1-driven tumorigenesis.

## Discussion

CRC is frequently identified at an advanced stage, reducing the number of treatment alternatives. Understanding heterogeneous immune cells of TME in CRC is more complex and crucial for successful cancer immunotherapy 3, 27. In developed nations, effective screening programs have led to a reduction in the incidence rates of CRC. We therefore employed scRNA-seq analysis of human colon tissues to characterize the functional heterogeneity of immune cell types such as T-cells, macrophages, and classical monocytes. Identifying prognostic factors in CRC is critical, with particular emphasis on the PD-1/PD-L1 interaction. Increased PD-L1 expression has been recognized as a critical factor linked to negative outcomes in CRC 28. Numerous studies indicate that PD-L1 expression in CRC is elevated and significantly correlates with clinicopathological characteristics and adverse prognostic outcomes, including diminished overall survival 10. For an instance, a study focused exclusively on serrated adenocarcinoma (SAC) patients within the CRC population revealed that one-quarter of these patients exhibited elevated PD-L1 expression and poor prognosis 29. Another study showed that expression of PD-L1 correlated with the prognosis of colorectal patients in terms of overall survival 30. Elevated levels of PD-L1 were linked to poor prognoses after surgery and correlated with increased expressions of TGF-β and Foxp3 in CRC patients 31, making PD-L1 a prospective therapeutic target for the treatment of CRC.

Given that the endogenous expression level of PD-L1 in CRC is a critical factor, screening for stabilizing agents or regulating PD-L1 protein abundance via the ubiquitin-proteasome system may serve as an alternative therapeutic approach for CRC. Few E3 ligases regulate PD-L1 levels through ubiquitination and degradation, thereby influencing immune therapies by obstructing immune checkpoints 32. FBXO22 is an E3 ligase that ubiquitinates the PD-L1 protein in cancer cells. The degradation of PD-L1 protein mediated by FBXO22 markedly enhances cancer immunotherapy by augmenting sensitivity to DNA damage-based treatments 33. HMG-CoA reductase degrading protein 1 (HRD1), an E3 ligase, is elevated in CRC. The HRD1 gene knock out led to the stabilization of mutant variants of PD-L1 protein, suggesting that HRD1 might be involved in PD-L1 degradation in CRC 34. STIP1 homology and U-box-containing protein 1 (STUB1) was known to destabilize PD-L1 protein by regulating lysine in the cytoplasmic domain 35. However, the role of DUBs in reversing PD-L1 ubiquitination, which may significantly contribute to PD-L1 stabilization and its correlation with CRC progression remains unexamined.

Several bodies of evidence indicate that DUBs regulate the stability of PD-L1 protein and are linked to various cancer progressions, including breast cancer, hepatocellular carcinoma, gastric cancer, bladder cancer, ovarian cancer, and pancreatic cancer 36. Nonetheless, the regulation of PD-L1 protein stability by these DUBs and its correlation with CRC remains unexplored. This study focused on investigating the expression level of DUB genes and PD-L1 in TME using a scRNA-seq dataset and its association with CRC progression. Here, we identified several putative DUBs that are correlated with PD-L1 expression in heterogeneous leukocytes of CRC (Figures 1-4). Among them, USP32 has emerged as a potential candidate for regulating PD-L1 protein levels (Figure 5), although there is currently no evidence supporting its association with CRC progression.

USP32 belongs to the USP family that regulates several cellular process, including DNA repair, cellular invasion, migration, and regulation of the cell cycle 37. Several reports indicate that USP32 functions as an oncogene and its expression is elevated across wide range of cancer types and linked to cancer progression 37, 38. For an instance, USP32 facilitates tumor immune escape in hepatocellular carcinoma 39, which is associated with the advancement of small cell lung cancer 40, breast cancer 41, glioblastoma 42, gastric cancer, and epithelial ovarian cancer 43. The silencing of USP32 has been shown to suppress gastric cancer tumorigenesis through the modulation of SMAD2 expression 44. USP32 also contributes to the development of YM155 drug resistance in breast cancers by downregulating SLC35F2 protein expression 22. Recent report suggest that USP32 promotes tumor progression by activating the RAF/MEK/ERK signaling pathway and inducing epithelial-mesenchymal transition (EMT) by stabilizing BAG3 protein expression in non-small cell lung cancer 45.

In this study, we examined *PD-L1* expression in various types of immune cells in human colorectal cancer in connection to cancer progression-specific USPs, including *USP10*, *USP12*, *USP14*, *USP18*, *USP32*, *USP33*, and *USP39*. We identified *USP32* as a uniquely expressed gene that is correlated with *PD-L1* expression in immune cell types of human colon cancer tissues, in contrast to human liver cancer tissues. Additionally, mouse scRNA-seq analysis revealed the significance of *USP32* expression in a PD-L1 knockout mouse model. Furthermore, we demonstrated that USP32 binds with, stabilizes, and prolongs PD-L1 half-life. Moreover, the knockout of the *USP32* gene in CRC hampered cell migration, invasion, proliferation, and colony formation in CRC cells. The ablation of the *USP32* gene led to a reduction in tumor development in a mouse xenograft study, suggesting that inhibition of USP32, which subsequently reduces PD-L1 protein levels, might be a viable therapeutic strategy for the treatment of CRC.

## Materials and Methods

### Dataset and sample information

For this study, we used two types of scRNA-seq datasets, including those from human and mouse datasets which were obtained from NCBI GEO datasets. For the qualitative and quantitative analysis, we obtained large datasets consist of 26 scRNA-seq samples from human (GSE231559) including 8 normal liver samples, 3 normal colon samples, 9 liver cancer samples, and 6 colon cancer samples. The mice (GSE246038) datasets consist of two groups: tumor colon cancer with PD-L1 wild type and tumor colon cancer with PD-L1 knockout, each containing more than two samples. All the samples consist of more than three files such as barcodes, genes and matrix files.

### Data processing

The collected datasets were preprocessed to obtain qualitative data, we used multiple AI integrated web-based tools such as Cellenics, Cytoanalyst and R Seurat package (5.3.0) to filter the datasets from transcriptomic of heterogeneous single cell types. Furthermore, to improve reliability and proficiency, we performed downstream analysis to ensure uniformity among all sample groups for further processing. During the pre-processing steps, we eliminated the dead cells through mitochondria unique molecular identifier (UMI) by setting gene number range between <550 or >6300. To eliminate doublets from all sample groups, the threshold was set to >0.5, resulting in high-quality datasets for each group. In this study, we used large datasets, therefore, to avoid the batch effect for subsequent analysis of sample group, we performed data integration. After pre-processing, we obtained 22831 genes from colon normal, 16938 genes from liver normal, 23412 genes from colon cancer and 23030 genes from liver cancer respectively. Similarly, for mice colon cancer with PD-L1 wild type group has 24189 genes and colon cancer with PD-L1 knockout group has 24930 genes. The dimensionality reduction was performed for each group and the data was recorded in Uniform Manifold Approximation and Projection (UMAP) by setting cosine distance metric 0.3.

### Cellular annotation

Automated cellular annotation was performed to screen the cell types by using ScType with reference marker gene tool developed by Lanevski et al. 46. The ScType database includes 4,212 cell markers for 194 cell types in 17 mouse tissues and 3,980 cell markers for 194 cell types in 17 human tissues. The marker genes database was constructed using PanglaoDB and CellMarker. Furthermore, the ScType specificity score for cellular annotation ensures that marker genes are constant across cell types and clusters, enabling high subpopulation selectivity and accurate unsupervised cell-type labeling. The ScType specificity score ranges from 0 to 1 (0 for non-specific markers, maximum occurrence; 1 for highly specific markers, minimal occurrence). “Unknown” cell types were defined as annotations with a negative ScType score and low confidence.

### Differentially expressed gene analysis

Following the cell annotation, the DEA was performed for each cell type and its subsets using the "Findmarkers" option from the R Seurat package. Based on DEA analysis, we explored the transcriptomic changes by identifying differentially expressed genes (DEGs), including both up and downregulated genes. Furthermore, string network analysis was used to uncover relationships between genes, regardless of whether they perform similar tasks or belong to related biological pathways.

### Gene ontology (GO) enrichment analysis

The GO was conducted for genome-wide USPs to ensure the functional annotation by using David annotation (https://davidbioinformatics.nih.gov/) and g:Profiler (https://biit.cs.ut.ee/gprofiler/gost). The GO was accomplished by entering the number of DEGs, which resulted in a list of candidate entities for biochemical processes, molecular functions and cellular components. The GO was completed by entering significant USPs, producing a list of potential entities for molecular functions, cellular components, and biochemical processes.

### Cell culturing condition and transfection methods

Human embryonic kidney (HEK293) (No. 21573), human colorectal carcinoma (HCT116) (No. 10247), and human colon adenocarcinoma cells (SW480) (No. 10228) are from the Korean Cell Line Bank (Seoul, South Korea). These cell lines are free from Plasmocin mycoplasma by Plasmocin treatment (Cat. No. ant-mpt1, InvivoGen). MycoAlert™ mycoplasma detection kit were used to detect mycoplasma content (Cat. No. LT07-118, Lonza Bioscience). The cells were cultured in RPMI and DMEM along with 10% FBS and 1% penicillin and streptomycin (Gibco BRL, Rockville, MD, USA) at 37 °C in a humidified atmosphere with 5% CO_2_. Every 3-4 days cells were passaged using 0.25% trypsin-EDTA. Cells were transfected with plasmids using polyethyleimine (PEI) (Polysciences, Inc. 24765) or Lipofectamine 3000 (Thermo Fisher Scientific, L3000001).

### Reagents and antibodies

Human PD-L1 (CSB-MA878942A) and USP32 (sc-374465) antibodies were used for western blot, immunofluorescence, and immunohistochemistry. GAPDH (sc-32233), normal mouse IgG (sc-2025), ubiquitin (sc-8017), HA (sc-7392), GFP (sc-9996), protein A/G Plus agarose beads (sc-2003), Ki67 (610969), Anti-Flag (M185-3 L), Alexa Fluor™ 488 (A21202), Alexa Fluor™ 594 (A21207), and protease inhibitor (11836153001). Lysis buffer (87787), cell lysis buffer (R2002), protein sample loading buffer (EBA-1052), cycloheximide (239765), MG132 (S2619), TAK243 (HY-100,487), PR-619 (ab144641), and puromycin (12122530) were used in study.

### Plasmids and sgRNAs

The pEGFP-N1/PD-L1 plasmid was purchased from Addgene (#121478). Truncated PD-L1 (1-260) lacking the ICD domain was sub-cloned into pEGFP-N1 vector. HA-USP32 and HA-USP32CA were kindly provided by Anja Bremm from the Institute of Biochemistry II, Germany. Truncations of USP32, U1 (1-366 aa), U2 (367-585 aa), and U3 (586-1604 aa) were sub-cloned into a pcDNA-HA tagged vector. Cas9-2a-mRFP-2a-PAC and sgRNA plasmids are from Toolgen, Korea. sgRNAs for *USP32* were selected using the CRISPOR tool and cloned as mentioned previously 17. Target sequence oligonucleotides were synthesized by Macrogen, Korea. The annealed oligonucleotides were then given terminal phosphates by T4 polynucleotide kinase. The target oligonucleotides were cloned into the sgRNA vectors, and the details of the primers are mentioned in Table S1.

### T7 endonuclease 1 assay

Genomic DNA was isolated using kits (Promega). For heteroduplex DNA, the nuclease target site-containing region of DNA was amplified by PCR. PCR amplicons are denatured at 95 °C and annealed to room temperature using water a bath. The annealed DNA was treated with T7E1 (New England Biolabs, USA) for 25 min at 37 °C and checked by electrophoresis. ImageJ software was used to quantify band intensity, and the evaluated mutation frequencies were recorded. The *USP32* PCR amplicon and cleavage sizes are mentioned in Tables S2 and S3.

### Quantitative real-time reverse transcription PCR (qRT-PCR)

RNA isolation and cDNA preparation were performed as described previously 23. The mRNA expressions of target gene mRNA were measured by normalizing with GAPDH as a control. The primers used for the qRT-PCR are shown in the Table S4.

### Immunoprecipitation assays

The transfected cells were lysed in IP lysis buffer for 25min, and protein estimation was done using Bradford reagent. 3 mg of cell lysate was treated with specific antibodies and incubated at 4 °C for 16 h. The next day cell lysates were incubated with 40 μL of protein agarose beads and kept for rotation at 4 °C for 4 h. The beads were then washed with lysis buffer and eluted in 2X SDS buffer. The samples were subjected to western blotting, and protein bands were detected by the ChemiDoc system. Mouse IgG (Cat# 31430) and rabbit IgG (Cat# 31460) were used as secondary antibodies for immunoblotting.

### Deubiquitination assay

HCT116 and HEK293 cells were used to determine USP32 DUB activity against endogenous and exogenous PD-L1 protein. MG132 (10 µM/mL for 6 h) was treated for 48 h post-transfection. The cells were lysed in a denaturing lysis buffer for 20 min. The antibodies were added to the cell lysates (2-3 mg) and incubated at 4 °C overnight. The next day, 40 μL of protein agarose beads were added and incubated for 3-4 h and washed with lysis buffer. The eluted samples in 2X SDS sample loading buffer were boiled for 5 min and analyzed by western blotting.

### Immunofluorescence staining

HCT116 cells cultured on glass coverslips at 37 °C in 5% CO_2_ incubator. The cells were washed with PBS, fixed using 4% paraformaldehyde, and finally permeabilized using 0.1% Triton X at 25°C for 8 min. Followed by wash and blocked with 3% BSA, the cells were incubated with the specific antibodies at 4 °C for 16 h. The washed cells were incubated with Alexa Fluor 488/594 antibodies for 1 h. DAPI was used to stain nuclei and mounted on a glass slide, and images were recorded.

### Duolink proximity ligation assay (PLA)

The binding between proteins was detected through the PLA kit (Cat. no. DUO92101). HCT116 and SW480 cells were fixed in 4% PFA and followed manufacturer's instructions. Cells were incubated with specific antibodies for 1 h incubation at 37 °C. Slides were washed three times and incubated with ligation ligase solution. The processed slides were treated with amplified polymerase solution and incubated in the dark for 100 min at 37 °C. Lastly, mounting medium containing DAPI was used to stain the cells, and images were recorded.

### Immunohistochemistry (IHC)

Mouse tumor tissue xenografts were embedded in paraffin and fixed with 4% PFA. Formalin-fixed paraffin-embedded tissues were taken for sectioning and stained with USP32 and PD-L1. The samples were counterstained with hematoxylin and then dehydrated. Finally, slides were mounted and images were recorded.

### Cell viability assay

HCT116 and SW480 cells (mock, USP32-KO, USP32-KO-reconstituted with USP32 or PD-L1) were seeded into 96-well plates and followed the protocol mentioned in the CCK-8 assay kit, and absorbance was recorded at 450 nm according to the manufacturer's instructions (Cat. No. CK04-11, Dojindo).

### Soft agar assay

HCT116 and SW480 cells (mock, USP32-KO, and USP32-KO-reconstituted with USP32 or PD-L1) were examined by colony formation assay. First, 35 mm culture dishes were plated with a 1:1 mixture of 1% agarose gel and 1X complete RPMI. The plates were then incubated for 16 h. Cells resuspended in 0.75% agarose with RPMI were cultured at a density of 0.1 × 10^5^ cells per well and incubated for 2 weeks. The colonies were stained with crystal violet dye (0.01%) diluted in 20% methanol and counted manually.

### Wound healing assay

A migration assay was used to examine migration behavior. HCT116 and SW480 cells (mock, USP32-KO, and USP32-KO-overexpressed with USP32 or PD-L1) were cultured. Using pipette tip, scratches were made in a specific pattern in the monolayers. Floating cells were removed using PBS wash, and the scratched cell layer was incubated at a 37 °C incubator. Migration of cells was recorded at 0 h and 24 h, and results were quantified.

### Invasion assay

Invasion assay was conducted using 0.8 μm matrigel coated Transwell chambers (Corning, NY, USA). HCT116 and SW480 cells (mock, USP32-KO, and USP32-KO-overexpressed with USP32 or PD-L1) were cultured at a density of 0.3 × 10^5^ cells per well. Next day, the cells on the upper surface of the insert were removed, and the cells at the bottom surface were fixed with cold methanol and stained with crystal violet. Cell invasion was recorded and quantified.

### Xenograft tumor experiment

NSG mice aged 5 weeks, were considered for xenograft tumor studies. This study was granted by the Hanyang University Institutional Animal Care and Use Committee. Mice were kept in a temperature-controlled room (12 h dark/light cycle and 55% relative humidity) with sufficient food and water. The experimental batch cells were resuspended in RPMI: Matrigel (1:1) and injected into each mouse. After 4 weeks, all mice were sacrificed by CO_2_ asphyxiation. The tumors were removed and recorded for its weight and volume.

### Statistical analysis

Graph Pad Prism 10.0 was used to conduct statistical analysis and graphical presentation. Results were calculated as the means and standard deviations from three independent trials. The variation between two groups was calculated using Student's t-test. One-way or two-way analysis of variance (ANOVA) was used to calculate experiments involving more than three groups, followed by Tukey's test. A P-value < 0.05 were regarded as statistically significant.

## Supplementary Material

Supplementary figures and tables.
