# Supplementary material for: Single-cell RNA sequence analysis reveals USP32 as a therapeutic target to mitigate PD-L1-driven colorectal tumorigenesis in vitro and in vivo
Source: Theranostics. 2026 Jan 1;16(2):986–1005. doi: 10.7150/thno.117900 (PMC12675009; doi:10.7150/thno.117900)
Supplement: Supplementary file 1 — Supplementary figures and tables. [file thnov16p0986s1.pdf]

Supplementary Information

Supplementary Figures:

Figure S1. Differentially expressed genes analysis in human colon cancer datasets.

Figure S2. Differentially expressed genes analysis in human liver cancer datasets.

Figure S3. UMAP visualization of PD-L1 expression.

Figure S4. The overall expression of PD-L1 gene in myeloid dendritic cells.

Figure S5. Functional annotation of differentially expressed USPs from human datasets.

Figure S6. Proposed molecular pathways of differentially expressed USPs from human datasets.

Figure S7. The population differences in PD-L1-WTand PD-L1-KO mice groups.

Figure S8. Differentially expressed genes analysis in mouse datasets.

Figure S9. Network analysis of total USPs.

Figure S10. Comparison of *Usp32* and PD-L1 gene expression in mouse across the cell clusters and significant cell types.

Figure S11. Functional annotation of differentially expressed USPs from mouse datasets.

Figure S12. The stabilizing effect of USP32 on PD-L1.

Figure S13. Stabilization effect of USP32 on ectopically expressing PD-L1 protein.

Figure S14. Generation of USP32 knockout clones in HCT116 and SW480 cells.

Figure S15. The effect of MG132 and TAK243 on PD-L1 protein level.

Figure S16. The effect of USP32 on ubiquitination of PD-L1 protein was graphically represented.

Figure S17. The effect of USP32 or PD-L1 on PD-L1 protein was graphically represented.

Figure S18. The loss of USP32 attenuates PD-L1-mediated carcinogenesis in SW480 cells.

Supplementary Tables

Table S1. Target sequences utilized for the production of sgRNA plasmids.

Table S2. PCR amplicon for the T7E1 assay is obtained using oligonucleotide sequences.

Table S3. Sizes of PCR amplicons and cleavage products following the T7E1 assay.

Table S4. Oligonucleotide sequences utilized for qRT-PCR**.**

Table S5. USP32 and PD-L1 mRNA expression scores derived from CCLE database.

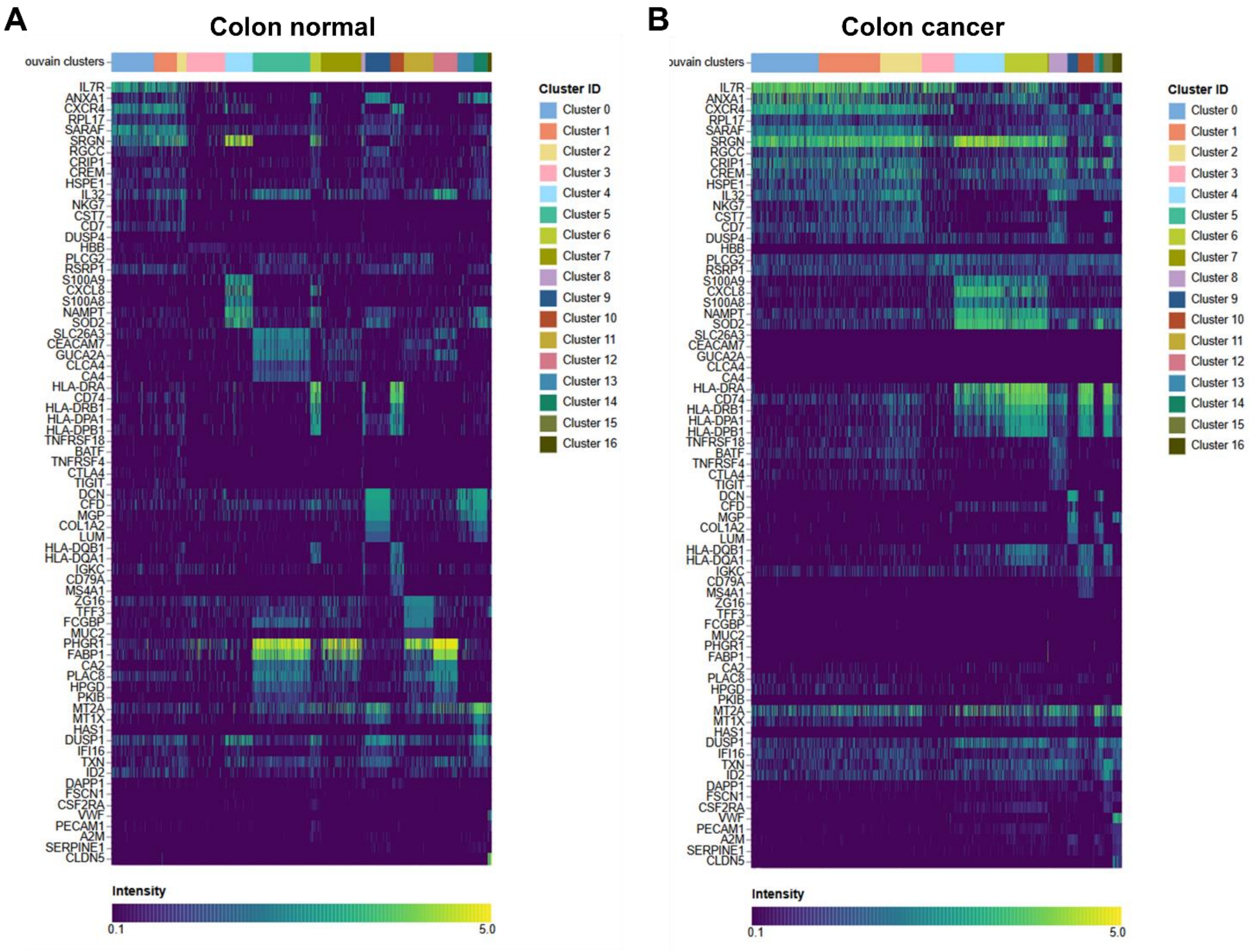

Figure S1. Differentially expressed genes analysis in human colon cancer datasets. The differentially expressed genes (DEGs) from heterogeneous clusters of both **(A)** the colon normal tissue and **(B)** colon cancer tissue were identified and visualized by Heat map expression analysis.

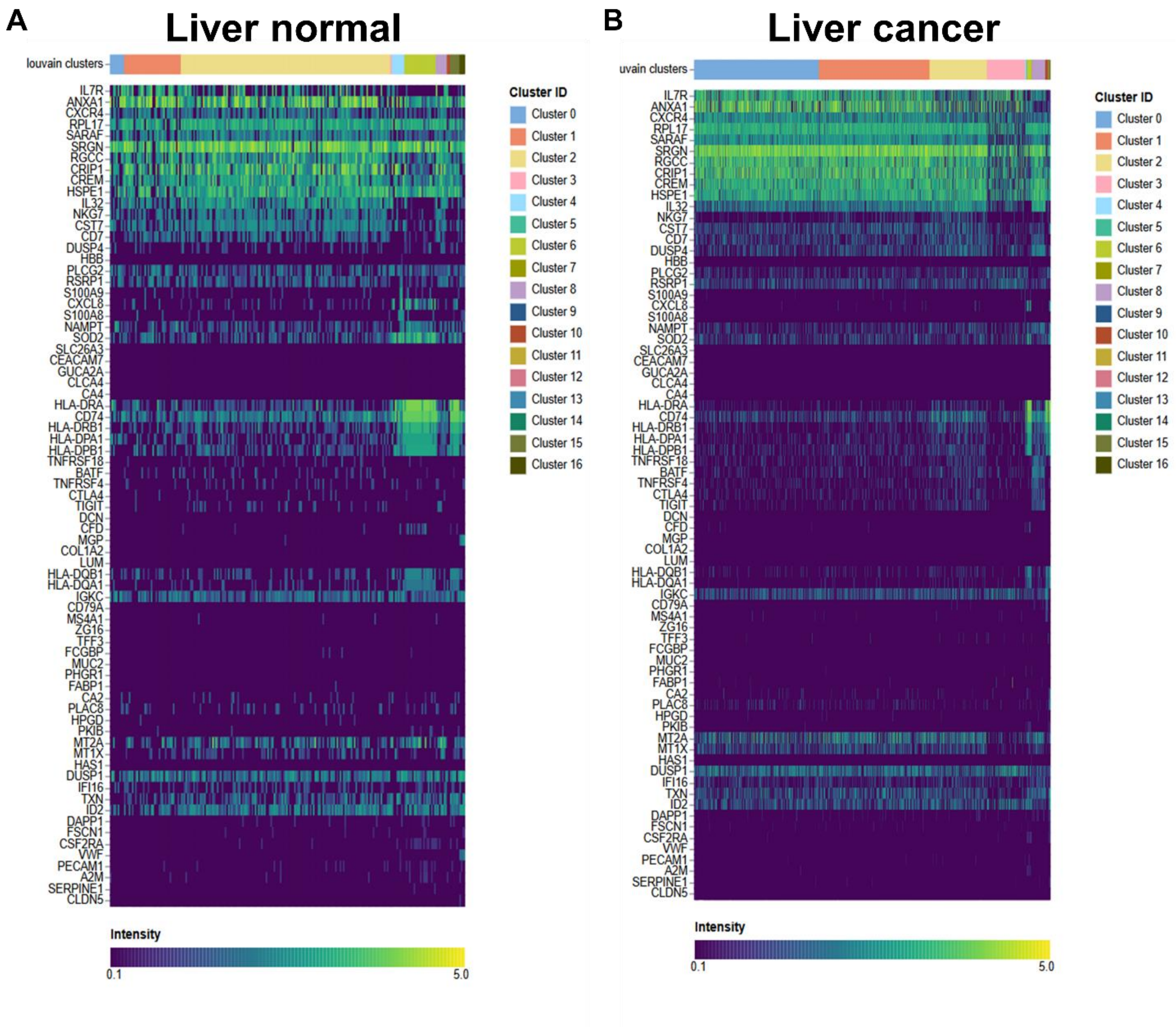

Figure S2. Differentially expressed genes analysis in human liver cancer datasets. The differentially expressed genes (DEGs) from heterogeneous clusters of both **(A)** the liver normal tissue and **(B)** liver cancer tissue were identified and visualized by Heat map expression analysis.

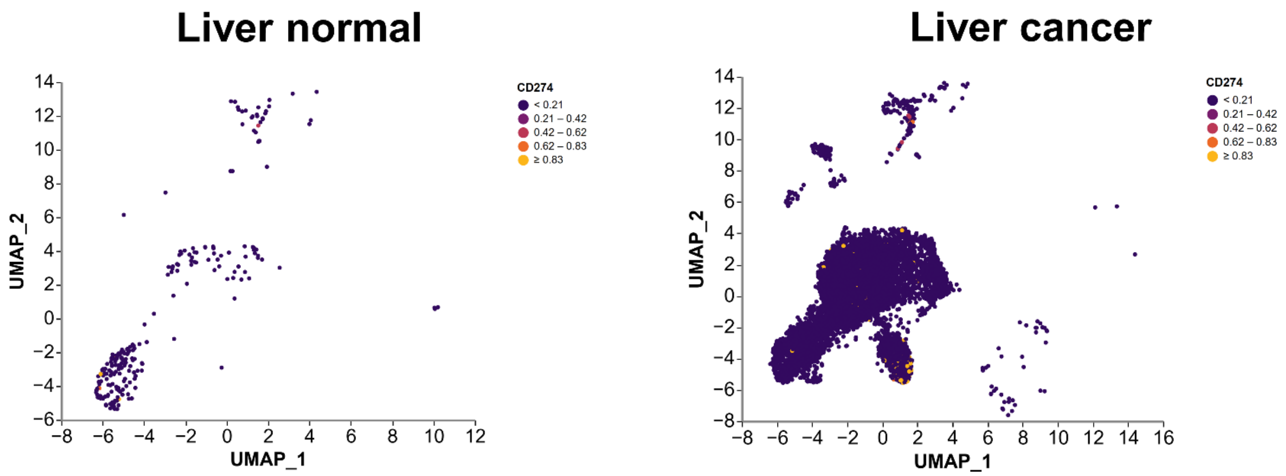

Figure S3. UMAP visualization of *PD-L1* expression. Comparison of *PD-L1* gene expression in both human liver tissue and liver cancer tissue and represented in UMAP.

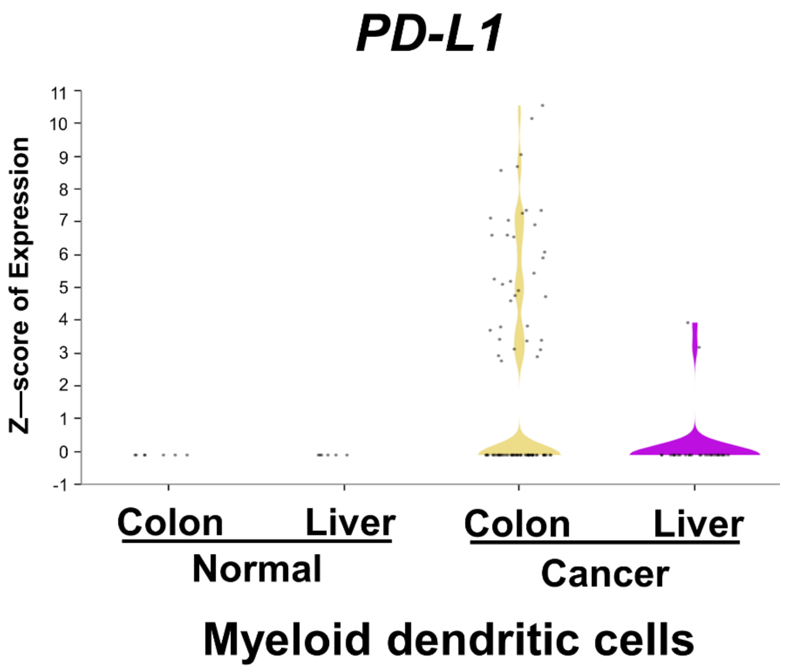

Figure S4. The overall expression of *PD-L1* gene in myeloid dendritic cells. Violin plot for comparisons of *PD-L1* level analysis expression in cancer tissues from colon or liver and its control tissue in myeloid dendritic cells.

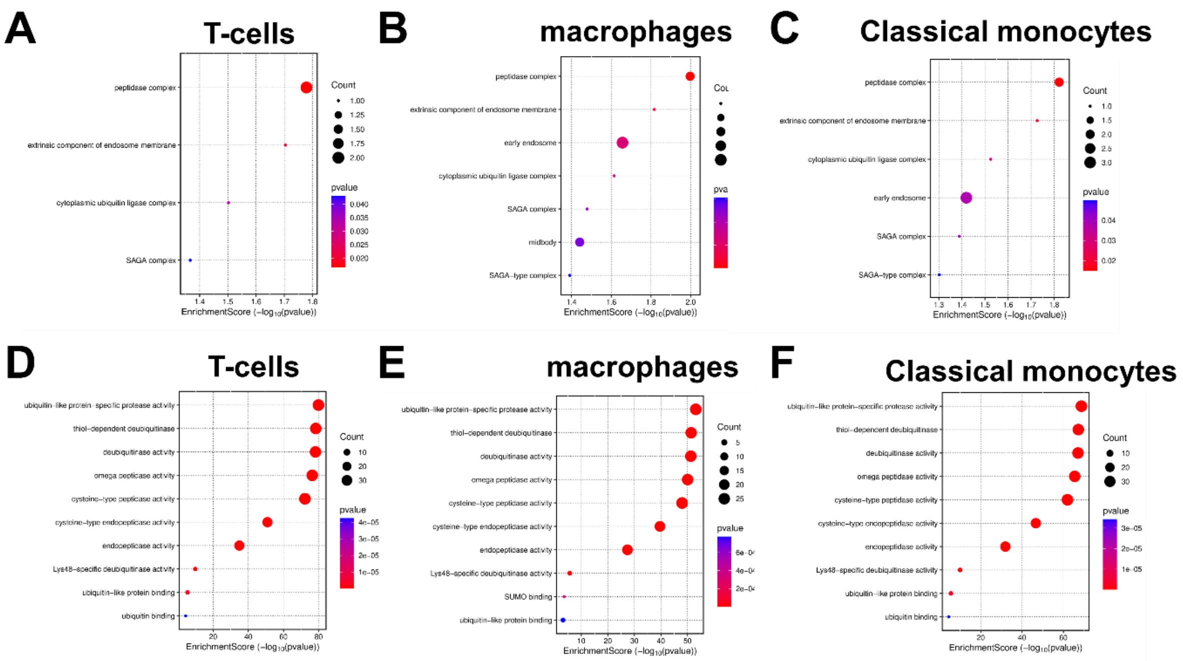

Figure S5. Functional annotation of differentially expressed USPs from human datasets. (A-C) Gene ontology performance particularly, cellular components for (A) T-cells, (B) macrophages and (C) classical monocytes were performed and visualized. **(D-F)** Gene ontology performance particularly, molecular function for (D) T-cells, (E) macrophage and (F) classical monocytes were performed and visualized.

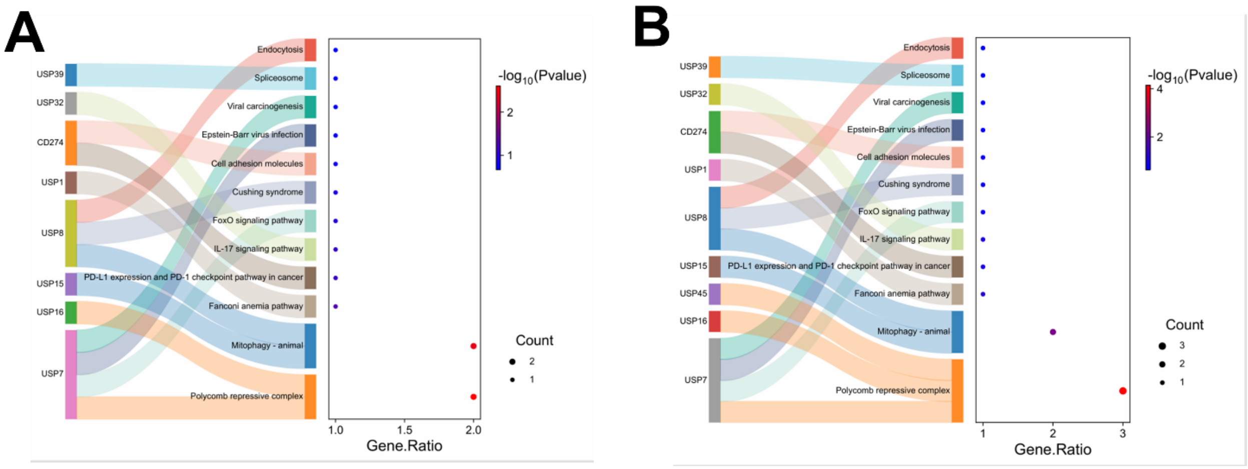

Figure S6. Proposed molecular pathways of differentially expressed USPs from human datasets. The Sankey and dot blot visualization predicted different molecular pathways of the differentially expressed USPs from **(A)** macrophage and **(B)** classical monocytes.

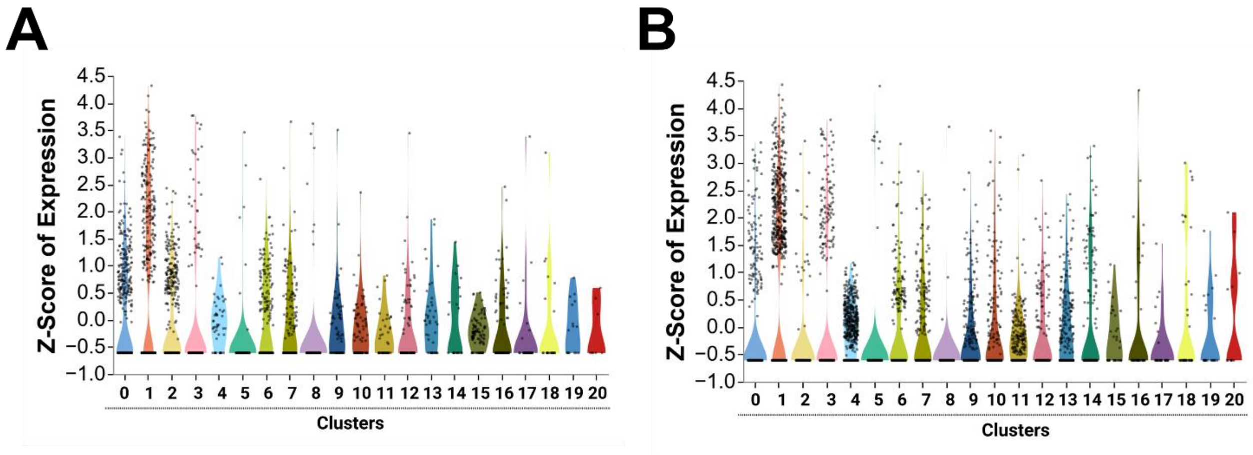

Figure S7. The population differences in PD-L1-WTand PD-L1-KO mice groups. The violin plot showed the population differences in total heterogeneous clusters from **(A)** PD-L1-WT and **(B)** PD-L1-KO mice groups

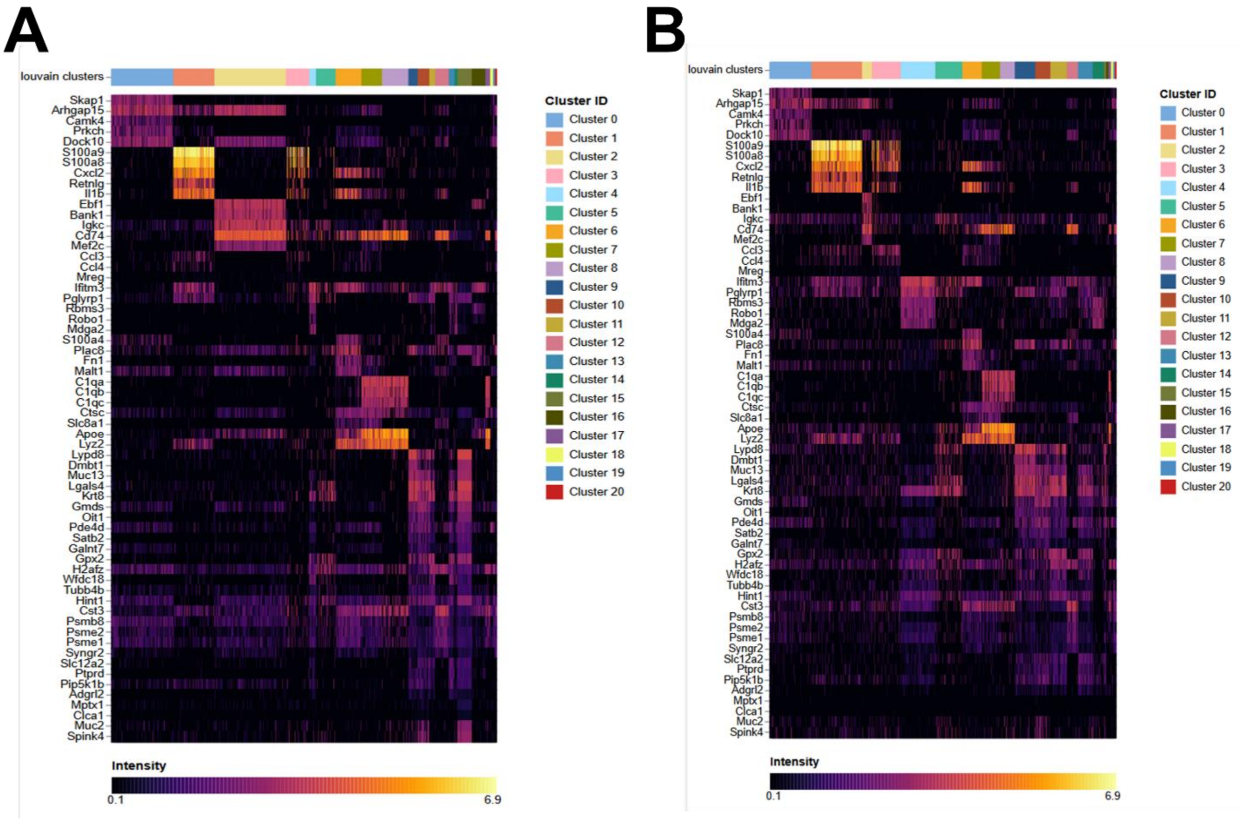

Figure S8. Differentially expressed genes analysis in mouse datasets. The differentially expressed genes (DEGs) from heterogeneous clusters of both **(A)** the PD-L1-WT and **(B)** PD-L1-KO mice groups were identified and visualized by Heat map expression analysis.

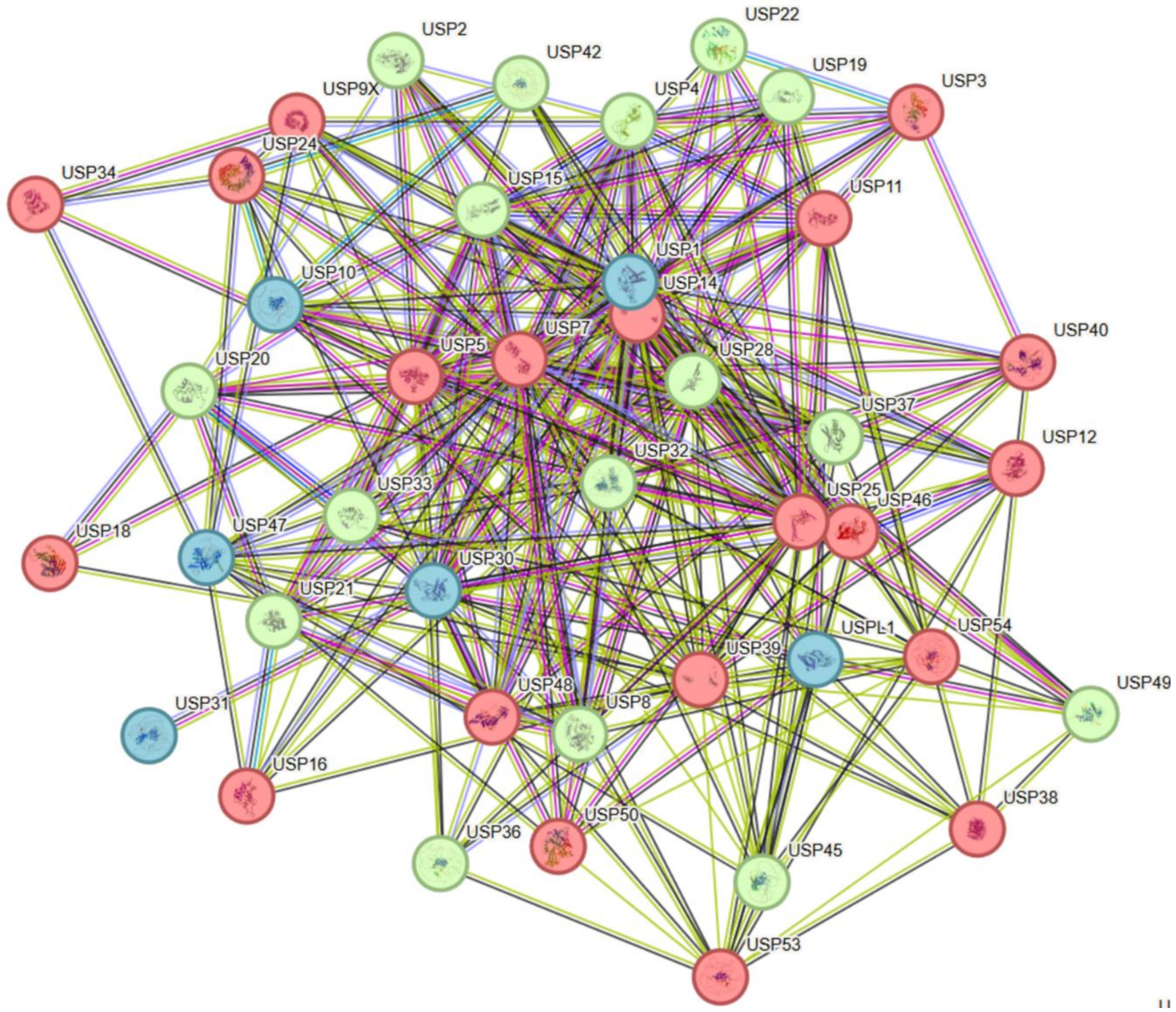

Figure S9. Network analysis of total USPs. Network analysis of total USPs from both human and mouse datasets were visualized by STRING database.

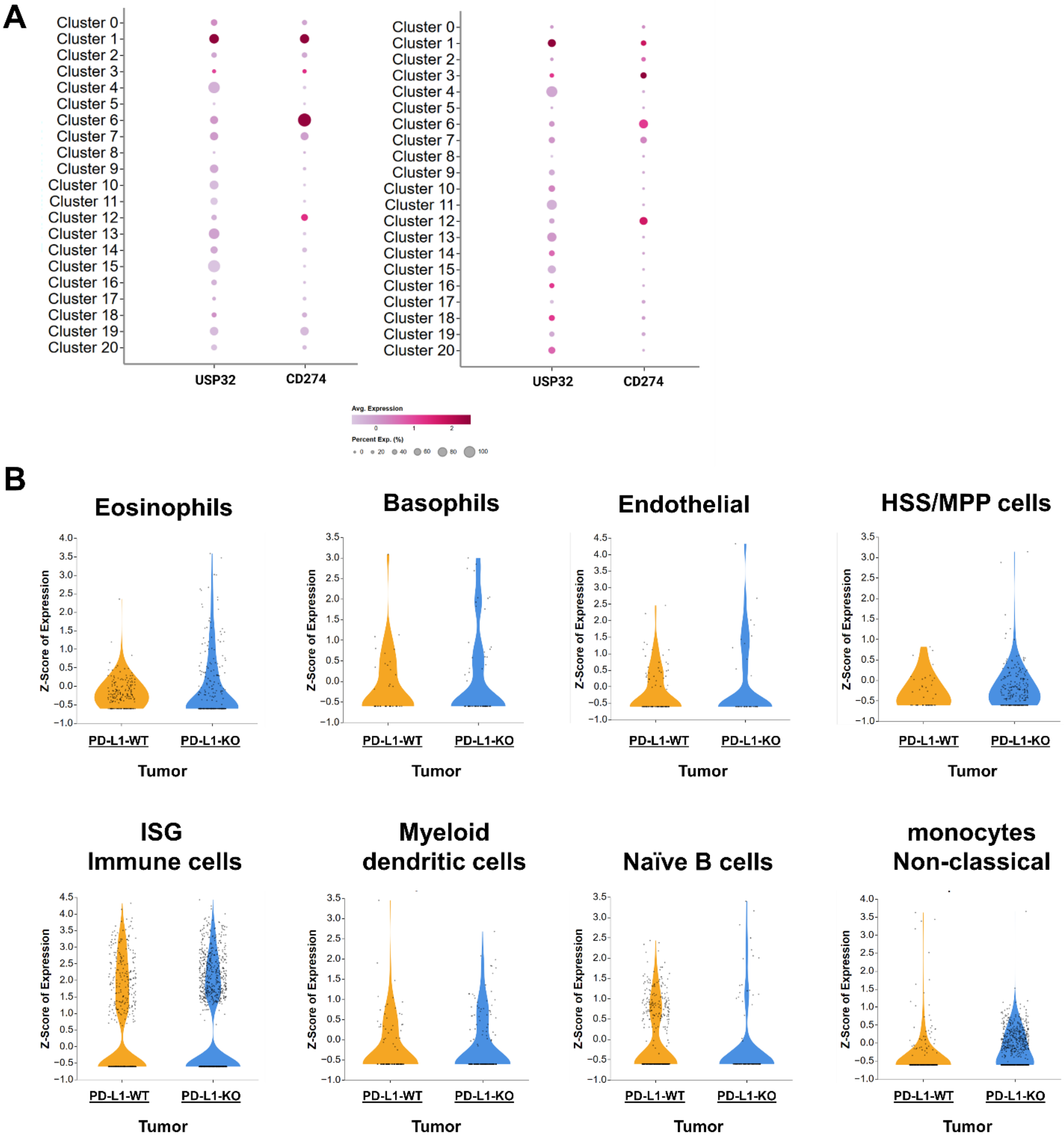

Figure S10. Comparison of *Usp32* and *PD-L1* gene expression in mouse across the cell clusters and significant cell types. (A) Dot-plot analysis revealed the expressions of both *Usp32* and *PD-L1* from more than 20 clusters. **(B)** Violin plot analysis showed the expression of *Usp32* in significant cell types compared between PD-L1-WT and PD-L1-KO mice groups

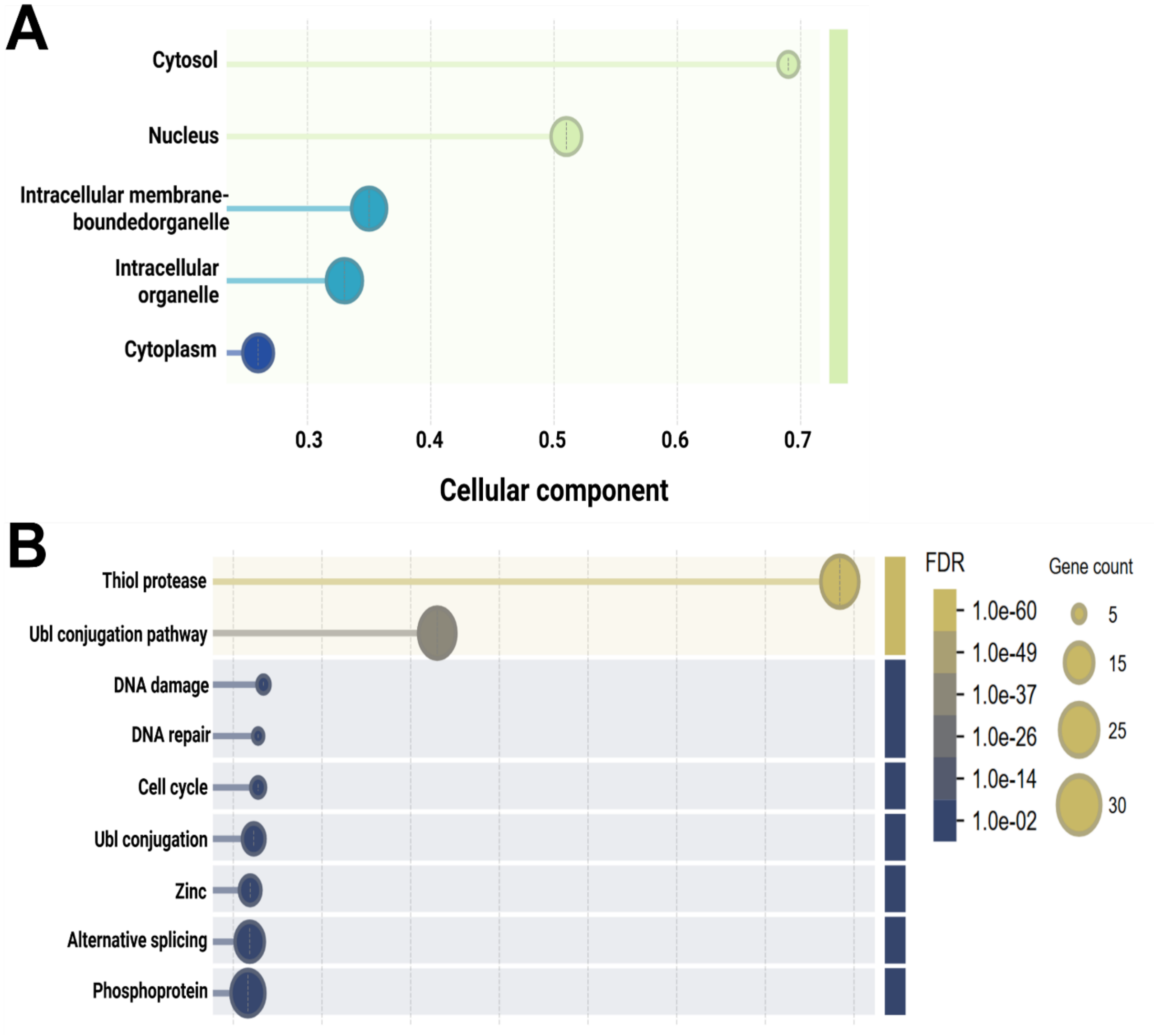

Figure S11. Functional annotation of differentially expressed USPs from mouse datasets. (A) Gene ontology analysis such as cellular components for USPs from the significant cell types were performed and visualized. **(B)** Similarly, gene enrichment analysis was performed for USPs from the significant cell types.

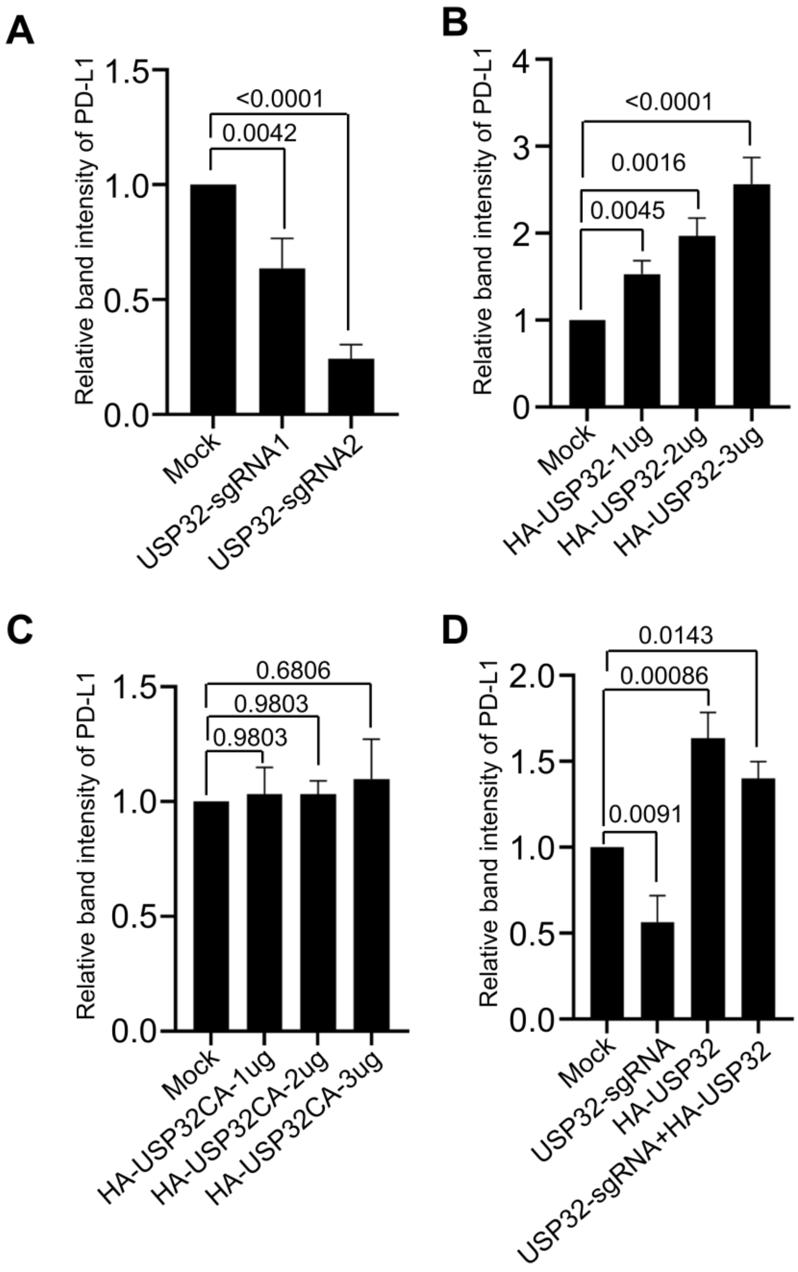

Figure S12. The stabilizing effect of USP32 on PD-L1. (A) In HCT116 cells, the impact of sgRNA targeting USP32 on the PD-L1 protein level was examined. **(B-C)** The stabilizing effect of (B) USP32 and (C) USP32CA. **(D)** The impact of HA-USP32 overexpression on PD-L1 protein in USP32-depleted HCT116 cells.

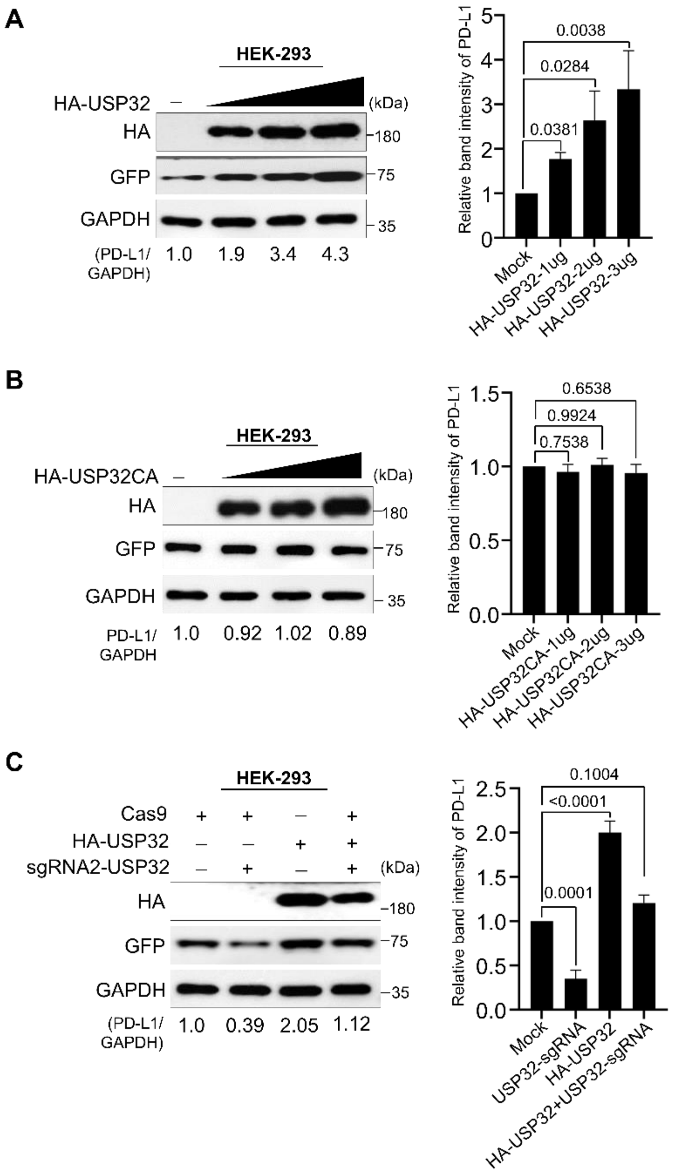

Figure S13. Stabilization effect of USP32 on ectopically expressing PD-L1 protein. (A) Increasing concentration of HA-USP32 or **(B)** HA-USP32CA transfected to assess exogenous GFP expressing PD-L1 protein. **(C)** The overexpression of HA-USP32 on exogenous PD-L1 protein in USP32-depleted HCT116 cells.

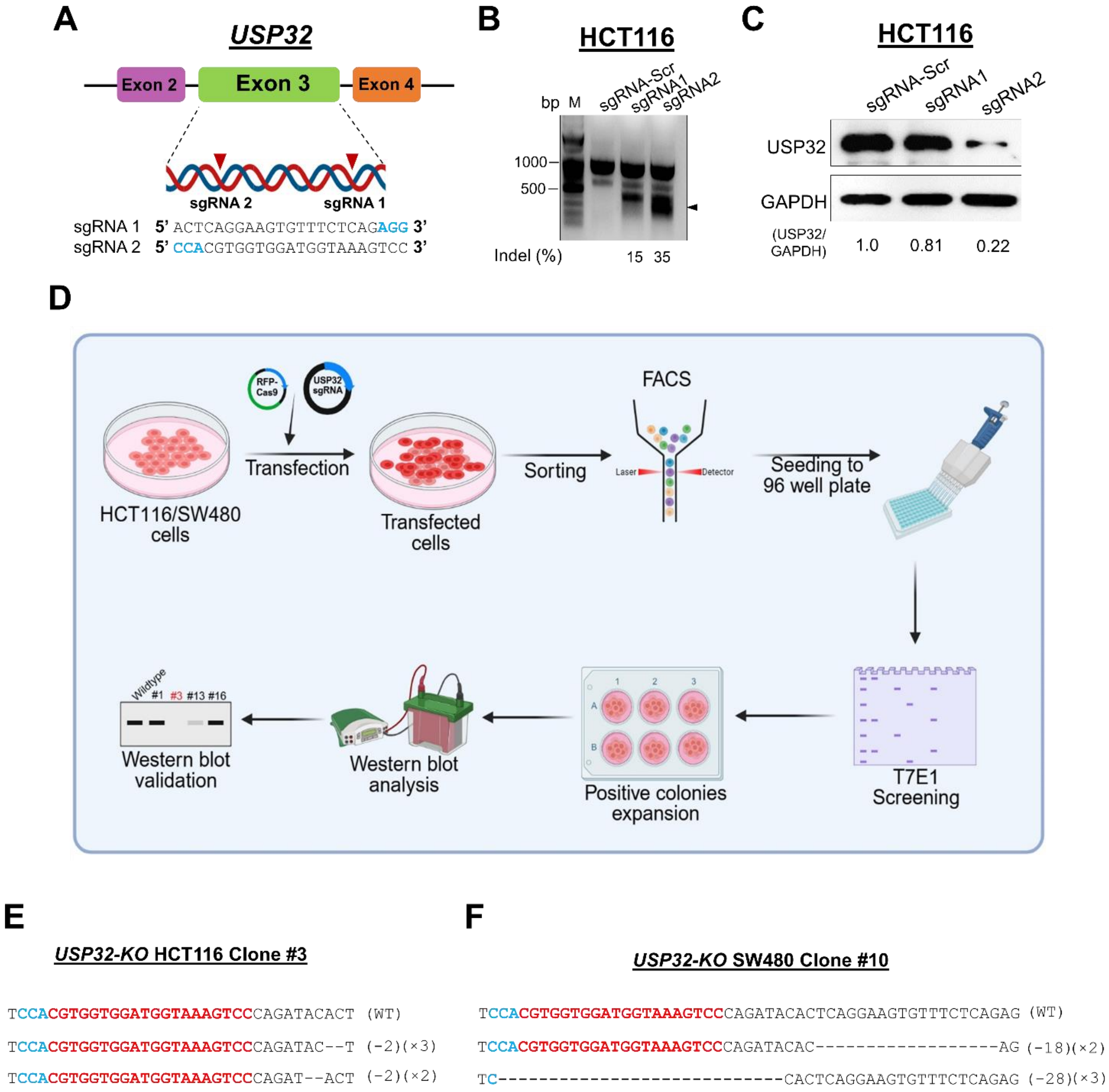

Figure S14. Generation of *USP32* knockout clones in HCT116 and SW480 cells. (A) The schematic representation of the sgRNA design approach at *USP32* gene exon 3. PAM sequences are shown in blue, while sgRNA sequences are shown in black colors (sgRNA1) (sgRNA2). **(B-C)** The cleavage efficiency of sgRNAs targeting *USP32* by T7E1 assay and (C) by western blot with USP32 antibody. **(D)** An overview of the workflow for

generating *USP32* gene knockout clones in HCT116 and SW480. **(E-F)** The *USP32* gene disruption in (E) HCT116 and (F) SW480 cells were confirmed by Sanger sequencing. The number of deleted or inserted bases are shown in parentheses.

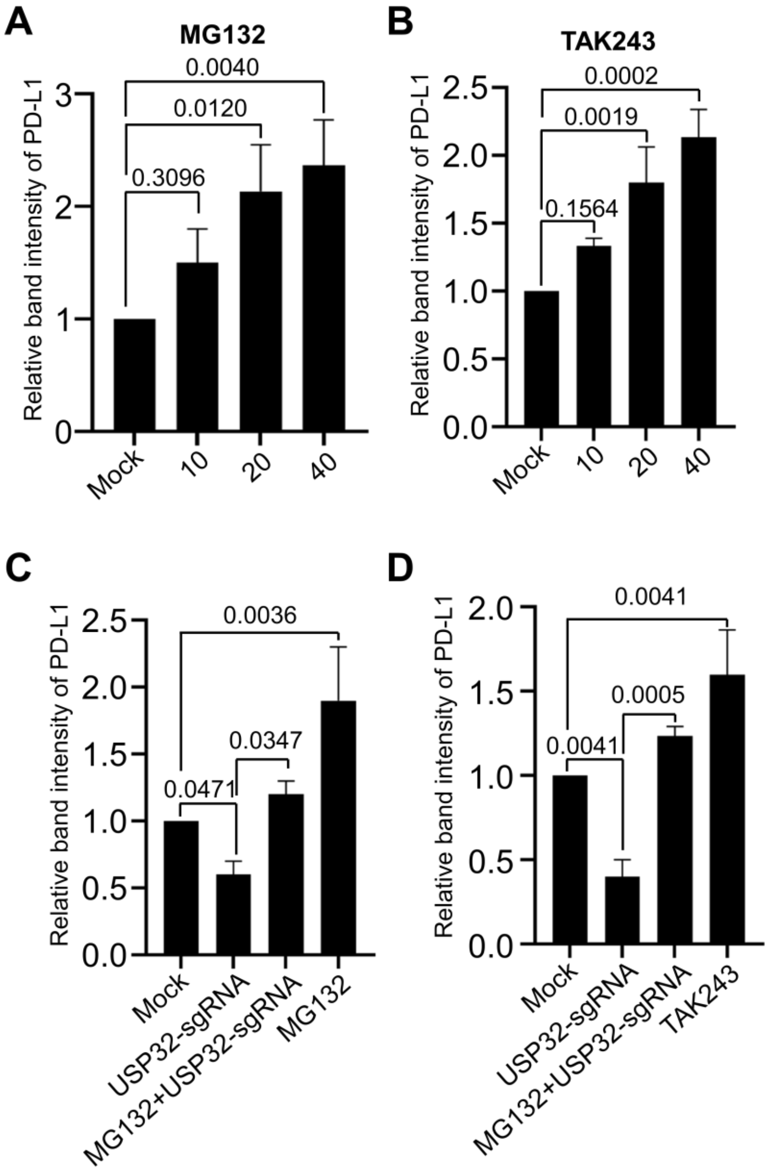

Figure S15. The effect of MG132 and TAK243 on PD-L1 protein level. (A-B) HCT116 cells were treated with MG132 and TAK243 for 6 h. **(C-D)** The impact of USP32 depletion on endogenous PD-L1 level in the presence of (C) MG132 (20 µM) and (D) TAK243 (20 µM). *P* values are indicated on the figures.

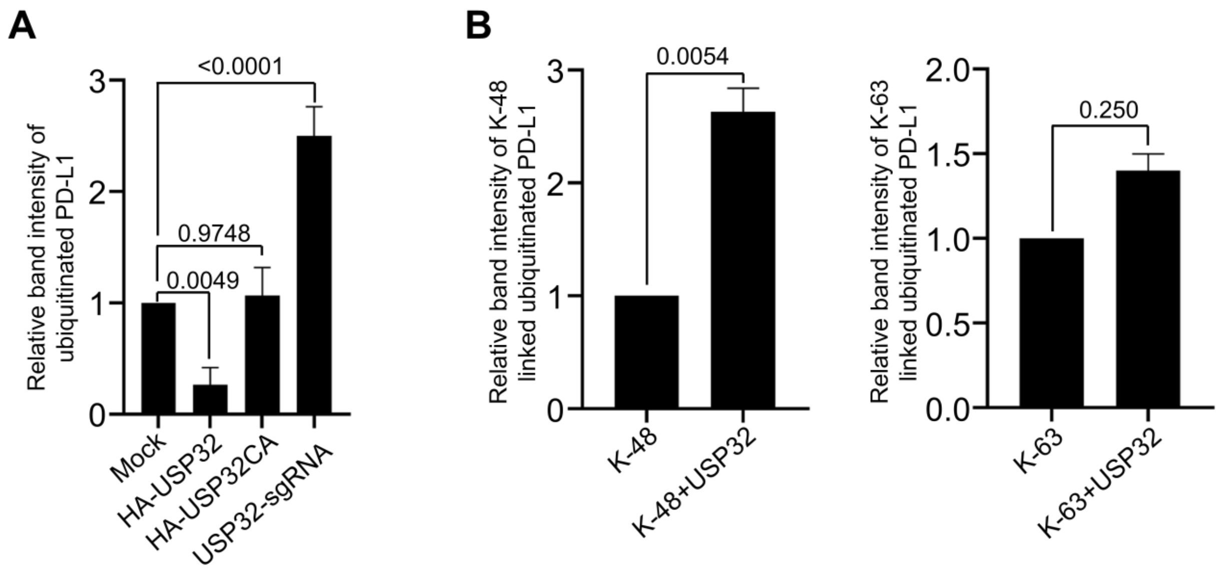

Figure S16. The effect of USP32 on ubiquitination of PD-L1 protein was graphically represented. (A) The ubiquitination status of endogenous PD-L1 was analyzed in the presence of USP32, USP32CA, and sgRNA targeting USP32 in HCT116 cells. **(B)** The effect of USP32 depletion on K-48 and K-63 linked polyubiquitination of exogenous PD-L1 protein by immunoprecipitation in 293T cells.

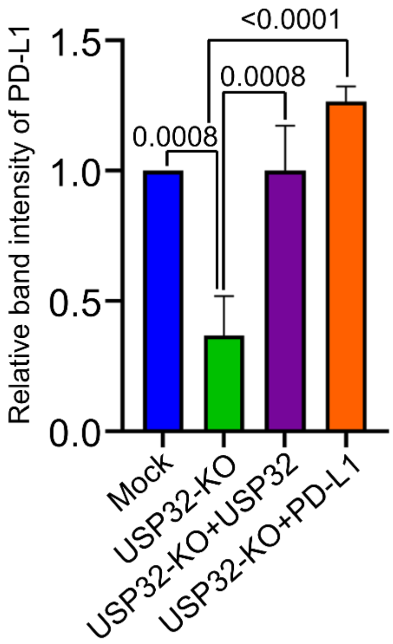

Figure S17. The effect of USP32 or PD-L1 on PD-L1 protein was graphically represented. The expression of USP32 and PD-L1 in mock, USP32-KO, and USP32-KO cells overexpressed with USP32 or PD-L1.

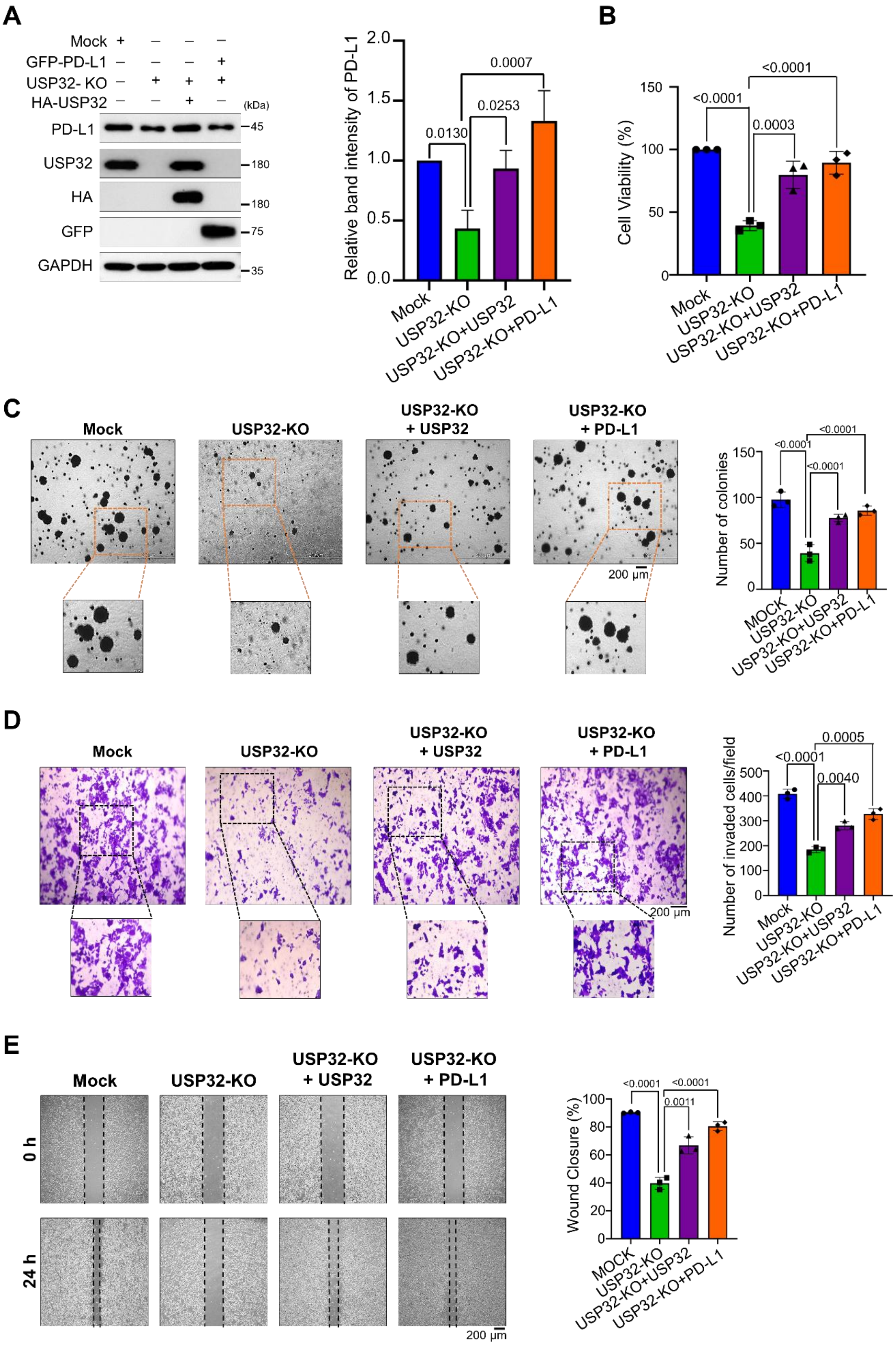
A
Mock
GFP-PD-L1
USP32- KO
HA-USP32
(kDa)
PD-L1
USP32
HA
GFP
GAPDH
45
180
180
75
35
Relative band intensity of PD-L1
0.0130
0.0253
0.0007
Mock
USP32-KO
USP32-KO+USP32
USP32-KO+PD-L1
B
Cell Viability (%)
<0.0001
0.0003
<0.0001
C
Mock
USP32-KO
USP32-KO + USP32
USP32-KO + PD-L1
200 μm
Number of colonies
MOCK
D
Number of invaded cells/field
0.0005
0.0040
E
0 h
24 h
Wound Closure (%)
0.0011

Figure S18. The loss of *USP32* attenuates PD-L1-mediated carcinogenesis in SW480 cells. (A) SW480 Mock, USP32-KO and USP32-KO cells overexpressed with USP32 or PD-L1 were analyzed by immunoblotting **(B-E)** The cells were subjected to (B) cell viability (C) colony formation (D) invasion and (E) migration, Scale bar = 200 μm.

Table S1. Target sequences utilized for the production of sgRNA plasmids.

Gene	sgRNA	Direction	Sequence (5' to 3')	Orientation
USP32	sgRNA1	FP	ACTCAGGAAGTGTTTCTCAGAGG	Sense
		RP	CCTCTGAGAAACACTTCCTGAGT	
	sgRNA2	FP	CCACGTGGTGGATGGTAAAGTCC	Anti-Sense
		RP	GGACTTTACCATCCACCACGTGG	

Table S2. PCR amplicon for the T7E1 assay is obtained using oligonucleotide sequences.

Gene	sgRNA		Direction	Sequence (5' to 3')
USP32	sgRNA1 and sgRNA 2	I PCR	FP	AGATGGAAGTGGAGTTTCTGGA
			RP	GTCACAGATGGCTCAAGGCTA
		II PCR	RP1	ACATGAGCACTGTTTCAGGTTC

Table S3. Sizes of PCR amplicons and cleavage products following the T7E1 assay.

Gene	sgRNA	PCR size	Cleavage size
USP32	sgRNA 1	876	435+379
	sgRNA 2	814	464+350

Table S4. Oligonucleotide sequences utilized for qRT-PCR**.**

Gene	Direction	Sequence (5' to 3')
USP32	FP	TGATGGAGTTCTCTCCAGGGT
	RP	TGACACACCTGGAAAAGGAGG
PD-L1	FP	CCTACTGGCATTTGCTGAACGCAT
	RP	ACCATAGCTGATCATGCAGCGGTA
GAPDH	FP	GTCATCCCTGAGCTGAACGG
	RP	CCACCTGGTGCTCAGTGTAG

Table S5. USP32 and PD-L1 mRNA expression scores derived from CCLE database.

**Cell line**	***USP32***	***PD-L1***
D458	7.494336	6.436462
SNU899	6.689159	4.877744
ISHIKAWAHERAKLIO02ER	5.680324	4.526695
CCLFHNSC0003T	5.612647	3.62293
UPCISCC200	5.413797	3.444932
YD15	5.288728	3.439623
KYSE150	5.207502	3.864929
SNU16	5.201242	3.743084
NP5	5.144862	4.185074
HEC116	5.111031	3.881665
HEC108	5.064366	3.893362

YD8	5.059615	3.253989
YD38	5.006298	3.166715
PFSK1	4.980025	3.892391
UPCISCC099	4.971773	3.025029
TGBC11TKB	4.971773	3.422233
OCUM1	4.929318	3.522307
DBTRG05MG	4.90641	3.989139
D283MED	4.888013	3.91265
NP8	4.885086	3.922198
538MGBA	4.862451	3.889474
CAL33	4.837943	3.044394
KON	4.831371	2.925999
TE5	4.827819	3.544733
NCCES1C1	4.786596	3.087463
NRHGCT2	4.783457	3.049631
BECKER	4.760753	3.835924
SAT	4.736064	2.807355
UPCISCC040	4.727376	2.784504
A673	4.727376	3.117695
SNGM	4.723012	3.559492
CCLFNEURO0005T	4.718088	3.743084
JEG3	4.715344	2.99458
OSC19	4.714795	2.797013
TC138	4.705978	3.01078
NUGC2	4.703211	3.201634
GSS	4.702103	3.214125
H103	4.667324	2.788686

SAS	4.662775	2.691534
KYSE520	4.657068	3.344828
SNU738	4.654206	3.746313
KOSC2	4.645586	2.729009
NUGC3	4.62527	3.095924
UPCISCC172	4.617063	2.648465
CCLFHNSC0001T	4.612352	2.627607
CCLFPEDS0010T	4.586164	2.893362
BICR3	4.547203	2.65764
ESO51	4.546586	2.980025
HEPG2	4.532317	2.536053
CCLFHNSC0004T	4.495056	2.510962
TE1	4.491853	3.179511
VAESBJ	4.491212	3.24184
SKGT2	4.487358	2.91265
SNU620	4.47703	3.060047
AN3CA	4.476382	3.341986
KATOIII	4.470537	2.950468
COLO680N	4.469235	3.15056
CHLA06ATRT	4.467932	3.423578
SNU1077	4.454176	3.350497
SNU216	4.441616	2.99458
CCLFOVPA0001T	4.420213	3.179511
RERFGC1B	4.415488	3.032101
KYSE180	4.415488	3.099295
KE39	4.414812	2.965323
UPCISCC026	4.412782	2.482848

EFE184	4.41007	3.294253
CCLFHNSC0002T	4.401903	2.411426
2313287	4.401221	2.857981
JHUEM1	4.398487	3.267536
CCLFUPGI0078T	4.393691	2.790772
SNU1076	4.38819	2.584963
CCLFNEURO0006T	4.3875	3.41819
HSC4	4.386811	2.592158
CHLA10	4.370862	2.742006
JHUEM2	4.356848	3.231125
PECAPJ34CLONEC12	4.354734	2.550901
SNU1214	4.354029	2.531069
HEC151	4.353323	3.193772
BT16	4.34979	3.329124
CCLFUPGI0005T	4.341986	2.763412
IM95	4.341986	2.809414
ANGMCSS	4.339137	3.399171
SNU668	4.321207	2.895303
ONDA8	4.319762	3.354734
D425	4.311067	3.253989
TDOTT	4.303781	3
SKNMC	4.300124	2.695994
MFE296	4.295723	3.174726
TC205	4.285402	2.589763
H376	4.275752	2.375735
MKN7	4.275007	2.799087
PECAPJ49	4.266787	2.438293

CHLA57	4.261531	3.206331
UPCISCC114	4.254745	2.301588
RDES	4.250962	2.646163
CCLFPEDS0008T	4.250204	3.001802
KYSE510	4.247928	2.91265
MFE280	4.245648	3.157044
HOKUG	4.243364	2.508429
BHY	4.237258	2.438293
ONDA9	4.221104	3.253989
D341Med	4.219556	3.234195
HS746T	4.215679	2.757023
TC71	4.211012	2.594549
BICR16	4.208673	2.422233
IS076A	4.203201	2.606442
TE9	4.201634	2.883621
FU97	4.19928	2.737687
UW228	4.190615	3.127633
HSC2	4.189825	2.411426
ONS76	4.171527	3.153805
UPCISCC090	4.165912	2.313246
SCC25	4.161081	2.408712
UPCISCC111	4.145677	2.195348
SCC15	4.144862	2.38405
MFE319	4.139961	2.969012
HSC3	4.135863	2.295723
EWS502	4.132577	2.518535
HEC50B	4.130931	3.014355

TASK1	4.121844	3.035624
OANC1	4.117695	2.531069
EMTOKA	4.117695	2.929791
UPCISCC072	4.100137	2.157044
CCLFNEURO0046T	4.086614	2.488001
T3M5	4.070389	2.094236
CCLFPEDS0007T	4.047887	2.356144
OE19	4.047887	2.735522
NP2	4.033863	3.087463
HEC6	4.032982	2.867896
SCC4	4.025915	2.266037
GSU	4.019702	2.570463
SH10TC	4.017031	2.508429
COGAR359	3.999098	2.93546
HHUA	3.991862	2.761285
BICR22	3.97728	2.134221
SNU638	3.975447	2.397803
HIRSBM	3.973611	2.735522
SNU520	3.969012	2.443607
KYSE70	3.958843	2.632268
HEC59	3.958843	2.778209
KYSE410	3.943921	2.615887
LN18	3.942984	3.030336
UPCISCC029A	3.927896	1.992768
SKNEP1	3.925999	2.263034
SF172	3.922198	2.998196
YD10B	3.916477	2.090853

HGC27	3.910733	2.389567
SW1783	3.869871	2.950468
MKN74	3.863938	2.375735
HEC1A	3.860963	2.709291
NCIN87	3.848998	2.403268
MKN1	3.848998	2.411426
JAR	3.844988	2.124328
UPCISCC131	3.837943	1.87578
SCC9	3.823749	2.07382
TE15	3.81455	2.536053
TB096	3.776104	2.801159
A253	3.773996	1.944858
RL952	3.770829	2.613532
CAL27	3.754888	1.906891
AGS	3.738768	2.217231
U251MGDM	3.730096	2.790772
CHLA99	3.722466	2.07382
SW579	3.69933	1.952334
JHUEM7	3.69933	2.526069
CADOES1	3.697107	2.084064
PECAPJ41CLONED2	3.68594	1.85997
OSC20	3.680324	1.752749
TE4	3.680324	2.324811
SKES1	3.667892	2.056584
TE11	3.667892	2.370164
NO10	3.667892	2.726831
DETROIT562	3.664483	1.910733

HEC1B	3.662206	2.526069
KYSE270	3.661065	2.316146
HEC265	3.65764	2.516015
TE10	3.634593	2.367371
TE14	3.619413	2.298658
BT12	3.619413	2.627607
TEN	3.613532	2.508429
H314	3.609991	1.718088
EW8	3.605257	1.9855
SNU1750	3.605257	2.025029
LNZ308	3.602884	2.695994
CHLA9	3.577731	1.937344
SKPNDW	3.536053	1.85599
170MGBA	3.521051	2.548437
JHUEM3	3.518535	2.430285
COLO684	3.513491	2.395063
KYSE450	3.500802	2.157044
SNU719	3.468583	1.944858
BICR31	3.463361	1.709291
FLO1	3.458119	1.883621
OE33	3.447579	2.007196
RPMI2650	3.44228	1.704872
SKGT4	3.432959	1.85599
OE21	3.432959	2.134221
ONDA7	3.432959	2.469886
SNU1041	3.431623	1.627607
UCSFOT1109	3.426265	1.443607

HSQ89	3.411426	1.432959
KYSE140	3.407353	2.07382
HUH6	3.403268	1.411426
HTST	3.395063	1.691534
SNU685	3.392317	2.283922
EN	3.381283	2.217231
TY82	3.357552	1.618239
ESO26	3.350497	1.786596
IS076P	3.336283	1.748461
SUMB002	3.295723	2.235727
KLE	3.270529	2.179511
HTMMT	3.258519	2.02148
CHLA32	3.249445	1.613532
NP3	3.244887	2.286881
BICR56	3.201634	1.361768
3.193772	4.335245	-1.14147
H413	3.189034	1.286881
UPCISCC116	3.137504	1.176323
CBAGPN	3.132577	1.510962
GI1	3.132577	2.226509
TE8	3.109361	1.823749
HEC1	3.099295	1.903038
PECAPJ15	3.085765	1.280956
SNU1	3.084064	1.550901
ECC10	3.077243	1.62293
H157	3.065228	1.182692
HT1080	3.056584	1.339137

ECC12	3.032101	1.641546
KYSE30	3.030336	1.709291
SNU5	3.01614	1.604071
JHESOAD1	2.989139	1.604071
CA922	2.970854	0.992768
DAOY	2.891419	1.87578
TE6	2.861955	1.555816
UPCISCC152	2.819668	0.956057
CHLA218	2.767655	1.137504
COGE352	2.744161	1.084064
HEC251	2.733354	1.550901
T3M3	2.731183	1
HUG1N	2.715893	1.31034
BICR78	2.713696	0.83996
CX03	2.691534	1.443607
FADU	2.673556	0.823749
SNU46	2.594549	0.815575
NCCSTCK140	2.560715	1.189034
SNU601	2.528571	1.049631
BICR6	2.508429	0.739848
NUGC4	2.500802	1.035624
H357	2.480265	0.584963
MKN45	2.454176	1.014355
TC106	2.395063	0.704872
UPCISCC074	2.386811	0.443607
HOUAI	2.375735	1.144046
UPCISCC154	2.367371	0.495695

JVE367	2.304511	0.584963
CHLA266	2.301588	1.257011
HO1U1	2.195348	0.214125
OACM51	2.182692	0.62293
SNU1066	1.948601	0.137504
ECGI10	1.847997	0.584963
MHHES1	1.778209	0.163499
LMSU	1.411426	0
